# Multimodal and longitudinal characterization of distinct tau and atrophy clusters in Alzheimer’s disease spectrum

**DOI:** 10.1038/s41598-025-98338-9

**Published:** 2025-05-25

**Authors:** Boris-Stephan Rauchmann, Ersin Ersözlü, Dorothea Luedecke, Nicolai Franzmeier, Robert Perneczky

**Affiliations:** 1 Department of Neuroradiology, LMU Hospital, LMU Munich, Munich, Germany; 2 Division of Mental Health of Older Adults, Department of Psychiatry and Psychotherapy, Ludwig-Maximilians-Universität München, Nußbaumstr. 7, 80336 Munich, Germany; 3 German Center for Neurodegenerative Diseases (DZNE) Munich, Munich, Germany; 4 Sheffield Institute for Translational Neuroscience (SITraN), University of Sheffield, Sheffield, UK; 5 Department of Psychiatry and Neurosciences, Charité Universitätsmedizin Berlin, Berlin, Germany; 6 German Center for Neurodegenerative Diseases (DZNE), Berlin, Germany; 7 Institute for Stroke and Dementia Research (ISD), LMU Hospital, LMU Munich, Munich, Germany; 8 Ageing Epidemiology (AGE) Research Unit, School of Public Health, Imperial College London, London, UK; 9 Munich Cluster for Systems Neurology (SyNergy), Munich, Germany

**Keywords:** Similarity-based Louvain clustering, Cognitive decline and dementia, Precision medicine, Phenotypical heterogeneity, Diseases of the nervous system, Biomarkers

## Abstract

Neuropathological and neuroimaging studies have identified several (endo-)phenotypes of Alzheimer’s disease (AD), suggesting a substantial heterogeneity in cerebral atrophy and tau spreading patterns. We included in our study a total of 320 participants, including healthy controls (N = 154) and patients across the AD spectrum (N = 166). We identified clusters of cerebral atrophy and tau PET uptake using a data-driven and similarity-based clustering approach, aiming to examine regional abnormality patterns in both modalities and differences in the clinical, cognitive, and biomarker characteristics among derived clusters. Abnormality patterns in tau PET and T1-weighted MRI within the same individuals revealed four distinct clusters for each imaging modality as surrogate markers of tau and neurodegeneration, respectively. The tau PET and atrophy clusters mainly showed substantial differences in their clustering allocations. While having the most severe biomarkers burden, the left temporal tau and diffuse atrophy clusters revealed the fastest clinical progression and steepest increase in tau PET uptake. Moreover, the diffuse atrophy cluster showed the fastest cortical volume loss, followed by the limbic-predominant atrophy cluster. Our results suggest characteristic differences between tau PET and atrophy clusters, especially for tau PET clusters, revealing more pronounced differences in cognitive profiles and disease biomarker trajectories than atrophy clusters.

## Introduction

Alzheimer’s disease (AD) is the most common neurodegenerative disorder^1^. Until recently, AD was seen as a clinically and neurobiologically relatively homogeneous disease entity. The AD-like tau deposition that has been understood as exhibiting a systematic staging (i.e. Braak stages)^2^ over the disease course, while the neurodegeneration (neuronal loss, i.e. atrophy) is not considered pathognomonic but associates closely with tau pathology^3–5^. However, the evidence of heterogeneity in AD is increasing, including a better understanding of possible neurobiological underpinning of heterogeneity^6–9^. Distinct AD “subtypes” have been identified recently^6,10,11^ in histopathological brain tissue analyses^12^ as well as tau positron emission tomography (PET) and structural magnetic resonance imaging (MRI) studies^13–22^. The reported subtypes were suggested to reflect underlying genetic, environmental, and neuropathological differences^6,11,23^. Our knowledge of the commonalities of subtypes in different modalities, including tau PET and MRI, is limited, even though an improved understanding of the manifestation of heterogeneity in AD would be important for developing precision diagnostics and treatment approaches^10,18^.

Data-driven methods such as Louvain community clustering analysis provide an unbiased approach, allowing the delineation of subtypes with a high replication rate^19^. In a previous study, we demonstrated the feasibility of this approach using the consensus method to identify four AD MRI-atrophy subtypes in two independent cohorts^24^. Cortical fibrillary tau protein spreading is associated with clinical severity in AD and was also identified as an important driver of neurodegeneration^25,26^, while its longitudinal trajectories need further exploration. Moreover, the characteristics in the variability of tau spreading patterns in AD have been described with high reproducibility rates among independent cohorts by scrutinizing the “typical AD” pattern, identifying a posterior and a left temporal pattern in addition to previously identified limbic-predominant (LP) and medial temporal sparing patterns^22^.

In this study, we adopted a more integrated approach than previous efforts. We identified clusters by analyzing cortical tau uptake and cortical atrophy in the same group of participants along the AD continuum, analyzing their spatial differences and overlaps and exploring their clinical, imaging and cognitive trajectories over time. We aimed for an improved understanding of the multimodal allocation to a particular cluster and the overlap and differences between the two underlying imaging biomarkers. Furthermore, we explored distinguishable characteristics between the derived clusters at baseline and in their longitudinal trajectories, considering imaging and non-imaging biomarkers as well as clinical severity and cognition. In the prevailing model of AD, it is hypothesized that neurodegeneration occurs after Amyloid-β (Aβ) and tau, with a presumed stronger relationship between neurodegeneration and tau pathology^27^. Building upon this premise, we set out to investigate the following hypotheses in our study: (1) Cluster allocation differs profoundly among modalities based on the known sequence of both pathologies during the natural course of AD. (2) Clusters within each modality show distinctly different characteristics in cerebrospinal fluid (CSF) biomarkers, clinical worsening, and cognitive decline in cross-sectional and longitudinal analyses.

## Results

### Group characteristics

The sociodemographic characteristics of the Alzheimer’s disease spectrum (ADS) and HC groups were well-matched, except for lower educational attainment in ADS. As expected, the ADS group comprised more frequently *APOE* ε4 allele carriers, had higher levels of CSF phosphorylated tau 181 (p-Tau) and total tau (t-Tau), lower levels of CSF Aβ42, lower cognitive composite scores in all four domains, higher tau- and Aβ PET tracer uptake, lower Mini Mental State Examination (MMSE) total score, higher Clinical Dementia Rating (CDR)-global score, and lower hippocampal volume (Table 1, eTable 1).

**Table 1:** Clinical, genetic and non-imaging biomarker characteristics of groups. Cognitive composite domain scores are presented in z-scores, while CSF biomarkers are in pg/ml. ^a^versus HC; ^b^versus Posterior/hippocampal-sparing; ^c^versus Limbic/Diffuse; ^d^versus MTL-sparing/minimal atrophy; ^e^versus left temporal/limbic-predominant. *Bonferroni-*p* < 0.001. †HC did not included in comparisons because its column proportion is equal to zero or one.

	HC (N = 154)		ADS (N = 166)		*p*	Tau clusters									Atrophy clusters								
						Posterior (N = 57)		Limbic (N = 53)		MTL-sparing (N = 36)		Left temporal (N = 20)		*p* (overall)	Hippocampal-sparing (N = 32)		Diffuse (N = 58)		Minimal atrophy (N = 41)		Limbic-predominant (N = 35)		*p* (overall)
	No	Mean/Frequency	No	Mean/Frequency		No	Mean/Frequency	No	Mean/Frequency	No	Mean/Frequency	No	Mean/Frequency		No	Mean/Frequency	No	Mean/Frequency	No	Mean/Frequency	No	Mean/Frequency	
Age, years	154	74 ± 6	166	75 ± 8	0.12	57	76 ^d^* ± 8	53	77 ^a,d^* ± 7	36	69 ^a,b^*^,c^* ± 8	20	74 ± 6	< 0.001	32	77 ^a,e^ ± 8	58	75 ± 8	41	74 ± 7	35	71 ^b^ ± 7	0.01
Years of Education, years	154	17 ± 2	166	16 ± 2	0.001	57	16 ^a^ ± 3	53	17 ± 2	36	16 ± 2	20	15 ^a^ ± 2	< 0.001	32	16 ± 3	58	16 ± 2	41	16 ± 3	35	16 ± 3	0.28
Sex, female	154	85 (55)	166	76 (46)	0.09	57	26 (46)	53	22 (42)	36	17 (47)	20	11 (55)	0.42	32	11 (34)	58	27 (47)	41	20 (49)	35	18 (51)	0.3
*APOE ε4* carrier	147	29 (20)	144	93 (65)	< 0.001	48	31 ^a^* (65)	47	27 ^a^* (57)	33	22 ^a^* (67)	16	13 ^a^* (65)	< 0.001	28	16 ^a^* (50)	49	28 ^a^* (57)	36	25 ^a^* (69)	31	24 ^a^* (77)	< 0.001
MMSE	147	28.95 ± 2.67	163	25.6 ± 4.01	< 0.001	56	26.61 ^a,e^ ± 3.08	52	25.94 ^a^* ± 3.81	35	24.89 ^a^* ± 4.32	20	23.1 ^a^*^,b^ ± 5.12	< 0.001	32	25.25 ^a^ ± 4.72	57	23.65 ^a^*^,d^*^,e^ ± 4.24	40	27.6 ^c^* ± 2.35	34	26.82 ^c^ ± 2.77	< 0.001
CDR-global score	147	0.03 ± 0.13	163	0.62 ± 0.37	< 0.001	56	0.55 ^a^* ± 0.28	52	0.62 ^a^* ± 0.42	35	0.64 ^a^* ± 0.33	20	0.75 ^a^* ± 0.5	< 0.001	32	0.61 ^a^*^,c^ ± 0.43	57	0.75 ^a^*^,b,d^*^,e^ ± 0.46	40	0.5 ^a^*^,c^* ± 0.2	34	0.53 ^a^*^,c^ ± 0.17	< 0.001
Diagnosis														0.18†									< 0.001†
MCI	154	0	166	112 (68)	< 0.001	57	41 (72)	53	39 (74)	36	22 (61)	20	10 (50)		32	23 (72)	58	25 (43)	41	36 ^c^* (88)	35	28 ^c^ (80)	
ADD	154	0	166	54 (33)	< 0.001	57	16 (28)	53	14 (26)	36	14 (39)	20	10 (50)		32	9 (28)	58	33 ^d^*^,e^ (57)	41	5 (12)	35	7 (20)	
MEM	152	1.04 ± 0.61	163	− 0.13 ± 0.78	< 0.001	56	0.08 ^a^*^,e^ ± 0.66	52	− 0.1 ^a^* ± 0.76	35	− 0.26 ^a^* ± 0.84	20	− 0.59 ^a^*^,b^ ± 0.88	< 0.001	31	− 0.14 ^a^*^,c^ ± 0.75	57	− 0.5 ^a^*^,b,d^*^,e^ ± 0.82	41	0.2 ^a^*^,c^* ± 0.63	34	0.08 ^a^*^,c^ ± 0.66	< 0.001
EF	152	1.18 ± 0.84	163	− 0.06 ± 1.14	< 0.001	56	− 0.02 ^a^* ± 1.08	52	0.13 ^a^* ± 1.16	35	− 0.18 ^a^* ± 1.12	20	− 0.46 ^a^* ± 1.22	< 0.001	31	− 0.14 ^a^* ± 1.14	57	− 0.66 ^a^*^,d^*^,e^ ± 1.12	41	0.44 ^a,c^* ± 0.86	34	0.42 ^a^*^,c^ ± 0.98	< 0.001
LAN	152	0.89 ± 0.68	163	-0.02 ± 0.99	< 0.001	56	0.09 ^a^*^,e^ ± 0.84	52	0.17 ^a^* ± 0.97	35	− 0.07 ^a^* ± 0.86	20	− 0.73 ^a^*^,b^ ± 1.32	< 0.001	31	0.02 ^a^*^,c^ ± 0.94	57	− 0.6 ^a^*^,b,d^*^,e^* ± 0.94	41	0.36 ^a,c^* ± 0.75	34	0.45 ^a,c^* ± 0.88	< 0.001
VIS	152	0.23 ± 0.67	163	− 0.24 ± 0.93	< 0.001	56	− 0.2 ± 0.93	52	− 0.19 ± 0.81	35	− 0.23 ± 1.11	20	− 0.46 ± 0.89	0.006	31	− 0.09 ± 0.89	57	-0.53 ^a^*^,d^* ± 1.01	41	− 0.05 ^c^* ± 0.75	34	− 0.17 ± 0.92	< 0.001
CSF p-Tau	72	17.81 ± 6.48	71	30.3 ± 17.86	< 0.001	30	30.96 ^a^ ± 18.5	16	28.27 ± 16.57	17	28.75 ± 10.07	8	35.21 ^a^ ± 30.13	< 0.001	12	34.97 ^a^ ± 13.22	21	32.17 ± 20.98	19	29.94 ± 21.82	19	25.65 ± 11.27	< 0.001
CSF t-Tau	72	209.66 ± 67.18	71	316.48 ± 164.24	< 0.001	30	309.03 ± 151.1	16	358.92 ^a^* ± 220.16	17	291.34 ± 87.47	8	312.93 ± 218.91	< 0.001	12	346.64 ^a^ ± 111.61	21	322 ^a^ ± 162.30	19	346.97 ± 235.08	19	260.53 ± 88.5	< 0.001
CSF Aß42	72	1768.4 ± 674.12	71	626.19 ± 303.11	< 0.001	30	722.42 ^a^* ± 332.14	16	643.56 ^a^* ± 267.8	17	510.84 ^a^* ± 137.23	8	475.71 ^a^* ± 407.94	< 0.001	12	693.78 ^a^* ± 304.14	21	612.69 ^a^* ± 286.04	19	609.11 ^a^* ± 323.96	19	615.51 ^a^* ± 318.13	< 0.001

*No* Number of available cases for the variable (please note that categorized variables have been indicated with numbers of all available cases for the given variable); *HC* Healthy controls; *ADS* Alzheimer’s disease spectrum; *MTL* Medial temporal lobe; *SD* Standard deviation; *MMSE* Mini-mental-state examination; *CDR* Clinical dementia ratio; *MEM* Memory; *EF* Executive function; *LAN* Language; *VIS* Visuospatial functioning; *p-Tau* Phosphorylated tau; *t-Tau* Total tau; *ROI* Region of interest; *Aβ42* Amyloid β 42.

### Characteristics of the tau and atrophy cluster spatial patterns

**Fig. 1:**
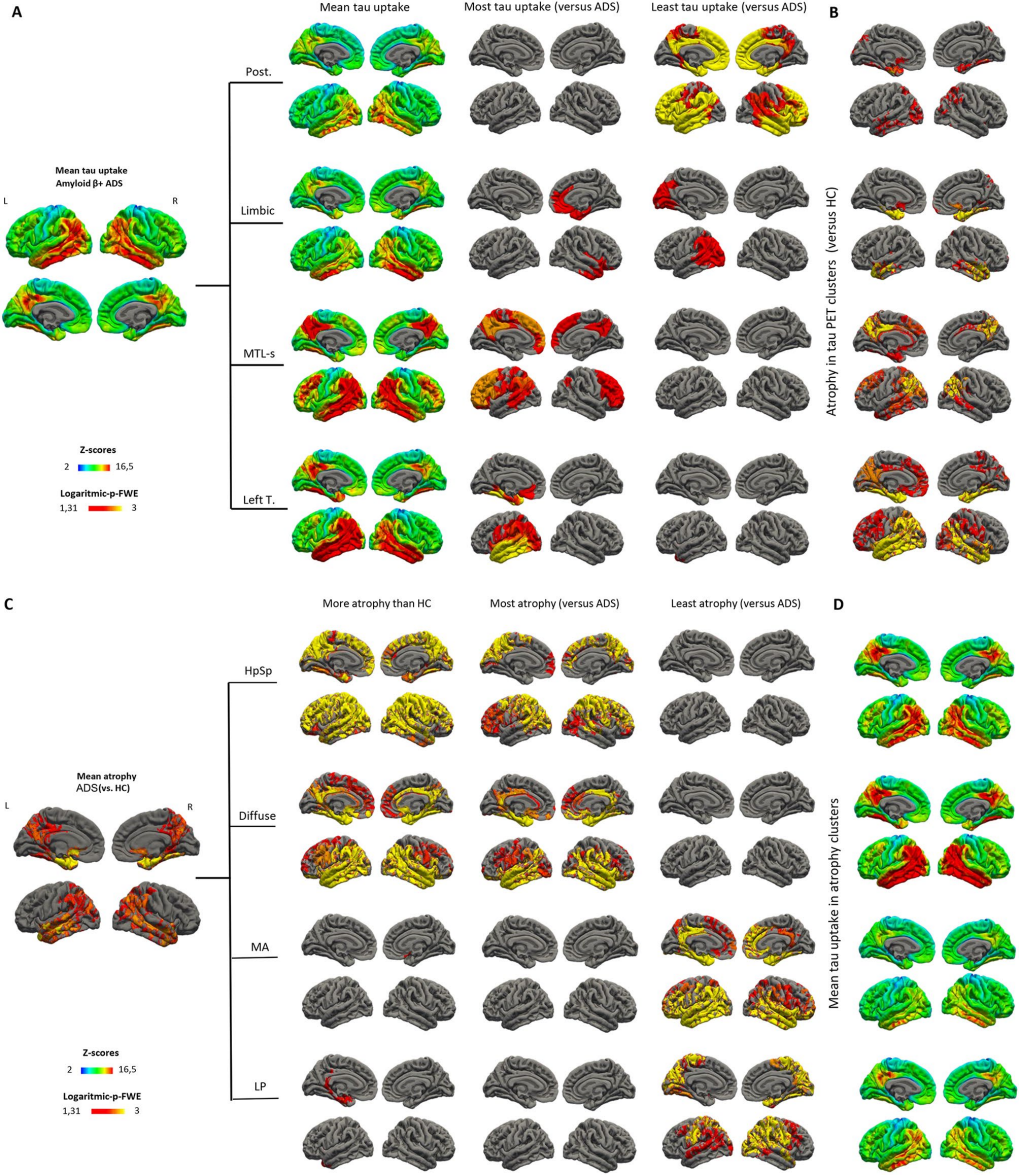
Tau and atrophy patterns of study groups are shown as mean tau PET uptake or following the censoring insignificant differences in cortical thickness, respectively. Mean tau uptake and mean atrophy (compared to HC) are additionally shown on the left on the group level for ADS. Of note, warmer tones represent increased abnormality (i.e. higher mean z-scores) in tau PET uptake, while higher cortical atrophy levels (i.e. higher logarithmic p) were masked for significant regions. (**A**) Group means of z-scores of tau uptake, most and least tau uptake compared to rest of the patients in ADS group. (**B**) The mean cortical atrophy compared to HC for tau PET clusters. (**C**) Mean cortical atrophy compared to HC, most and least atrophy compared to rest of the patients in ADS group. (**D**) Group means of z-scores of tau uptake for atrophy clusters. HC, healthy controls; ADS, Alzheimer’s disease spectrum; Post., posterior; MTL-s, medial temporal lobe sparing; Left T., left temporal; HpSp, hippocampal-sparing; MA, minimal atrophy; LP, limbic-predominant; FWE-p, family-wise error corrected p.

Through the clustering of patients along ADS using vertex-wise z-scores of cortical tau PET uptake, we identified the following clusters of tau PET binding patterns: (i) A posterior tau PET cluster with pronounced parietal and occipital tau binding, (ii) a limbic tau PET cluster with tau PET binding predominantly in the right medial temporal lobe, the insula, the orbitofrontal and anterior cingulate cortex and lower levels in parietal and occipital regions, (iii) a medial temporal lobe-sparing (MTL-sparing) tau PET cluster with tau binding in bilateral parietal and lateral-temporal and frontal regions (iv) a left temporal tau PET cluster with a left-lateralized temporal tau binding pattern which partly also covers parietal and occipital brain regions. Each tau cluster revealed distinct differences in tau deposition patterns when compared to all other tau clusters, illustrated as most and least tau regions (Fig. 1A) and pairwise comparisons between any tau PET clusters (eFigure 1A, B).

Through a separate clustering of vertex-wise z-scores of cortical thickness from structural T1-weighted MRI scans, we identified the following atrophy clusters: (i) A hippocampal-sparing (HpSp) atrophy cluster with atrophy patterns predominantly comprising the association cortices and relatively sparing the medial and temporal lobes and the hippocampus. The ratio of cortical volume to hippocampal volume (CTV:HV) was not reduced and comparable to HC (eTable 1), (ii) a diffuse atrophy cluster with higher cortical atrophy in both the limbic and neocortical regions and similar levels of temporal and parietal atrophy compared to controls, (iii) a minimal atrophy (MA) cluster with overall significantly less cortical atrophy, and (iv) a LP atrophy cluster with atrophy predominantly in limbic regions in comparison to HC (Fig. 1C). As for the tau PET clusters, we calculated statistical differences between the atrophy distribution patterns between each pair of atrophy clusters, reported in the supplementary material (eFigure 1C).

In a subsequent analysis, we analyzed the relationship between atrophy and tau clusters by exploring the underlying atrophy patterns in the tau PET-derived clusters and vice versa to gain insights into similarities and differences between both imaging biomarkers within the clusters. Within the tau PET clusters, the posterior tau PET cluster showed pronounced atrophy in the occipital and parietal regions, in the medial temporal lobe and the insula; the limbic tau PET cluster mainly in parietal, temporal and frontal brain regions; the MTL-sparing tau PET cluster pronounced in parietal and temporal areas; and the left temporal tau PET cluster in temporal and parietal brain regions (left > right) (Fig. 1B). Overall, the analysis of tau tracer uptake patterns within the atrophy-derived clusters revealed a relatively congruent pattern between modalities (Fig. 1D).

**Fig. 2:**
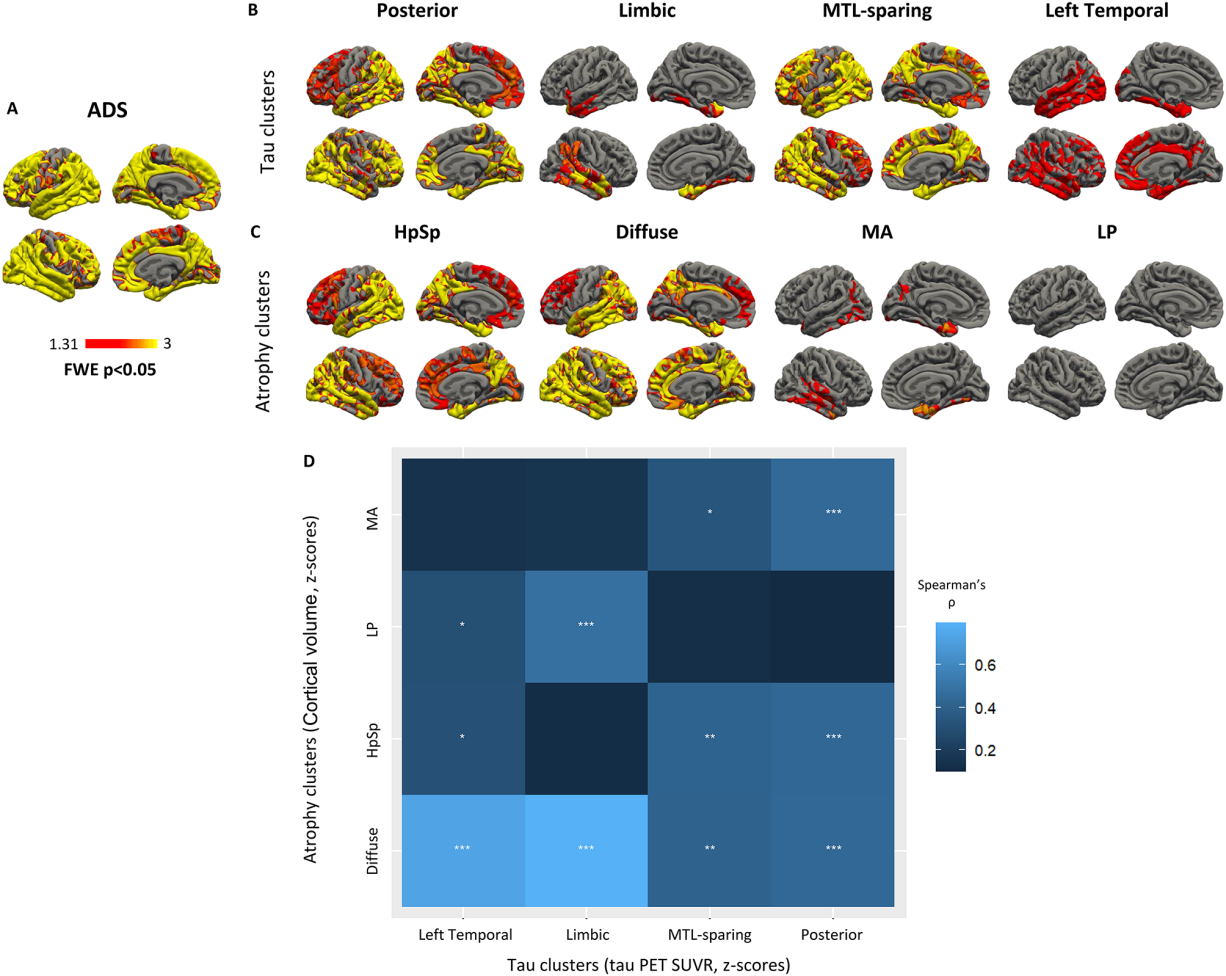
Vertex-wise correlations between tau uptake and cortical thickness in ADS cohort (**A**), within each tau cluster (**B**), and each atrophy cluster (**C**), including results with FWE-adjusted *p* < 0.05. (**D**) Heatmap of the correlation coefficients between the covariance in cortical volume within atrophy clusters and tau PET within tau clusters. *Bonferroni-p < 0.05, ** Bonferroni-p < 0.01, ***Bonferroni-*p* < 0.001. ADS, Alzheimer’s disease spectrum; MTL-sparing, medial temporal lobe sparing; Left Temp., left temporal; HpSp, hippocampal-sparing; MA, minimal atrophy; LP, limbic-predominant; FWE, family-wise error.

Next, we assessed how the atrophy distribution pattern within a cluster was associated with the tau distribution pattern, while we found an association between cortical atrophy and tau uptake in ADS in a widespread fashion (Fig. 2A). We tested vertex-wise associations between tau PET and cortical thickness z-scores within each cluster (Fig. 2B and C for tau PET and atrophy clusters, respectively). Our findings revealed close associations between both modalities in terms of the posterior and MTL-sparing tau clusters, as well as the HpSp and diffuse atrophy clusters. The key differences observed among the posterior and MTL-sparing tau clusters, as well as the HpSp and Diffuse atrophy clusters, primarily revolved around the symmetry of atrophy patterns and the extent of involvement in frontal and occipital regions. Interestingly, the limbic tau cluster revealed associations between both modalities in the right temporal lobe and the left temporal tau PET cluster in the left temporal lobe. Also, the MA cluster showed associations between both modalities in the temporal lobe with a right-sided dominance. Surprisingly, the LP atrophy cluster showed no significant correlation between tau uptake and atrophy.

### Overlap between imaging-derived clusters

We analyzed the overlap between cluster group allocation of participants in tau PET and atrophy-defined clusters. The results revealed a relative heterogeneity in allocation between the two modalities (eFigure 3), with varying levels of correlations in the spatial overlaps of variance for atrophy versus tau clusters (Fig. 2D).

In particular, we observed strong correlations between the regional tau covariance in both the limbic and left temporal tau PET clusters and the covariance in atrophy within the diffuse atrophy clusters (Spearman’s ρ = 0.79 and ρ = 0.73, respectively) (Fig. 2D and eFigure 2). Moreover, the limbic tau cluster also exhibited a moderate correlation with the LP atrophy cluster in their covariance patterns (Spearman’s ρ = 0.48). The HpSp atrophy cluster showed moderate correlations in covariance patterns with the MTL-sparing, posterior and left temporal tau clusters (Spearman’s ρ = 0.41 and ρ = 0.43, respectively) (Fig. 2D and eFigure 2). Furthermore, clusters did not differ significantly (p = 0.06) when compared by using chi-square test (eFigure 3). However, the highest frequency of shared cases has been observed in left temporal tau cluster, having 70% subjects who are also identified in diffuse atrophy cluster.

### Clinical, cognitive and genetic characterization of clusters

**Fig. 3:**
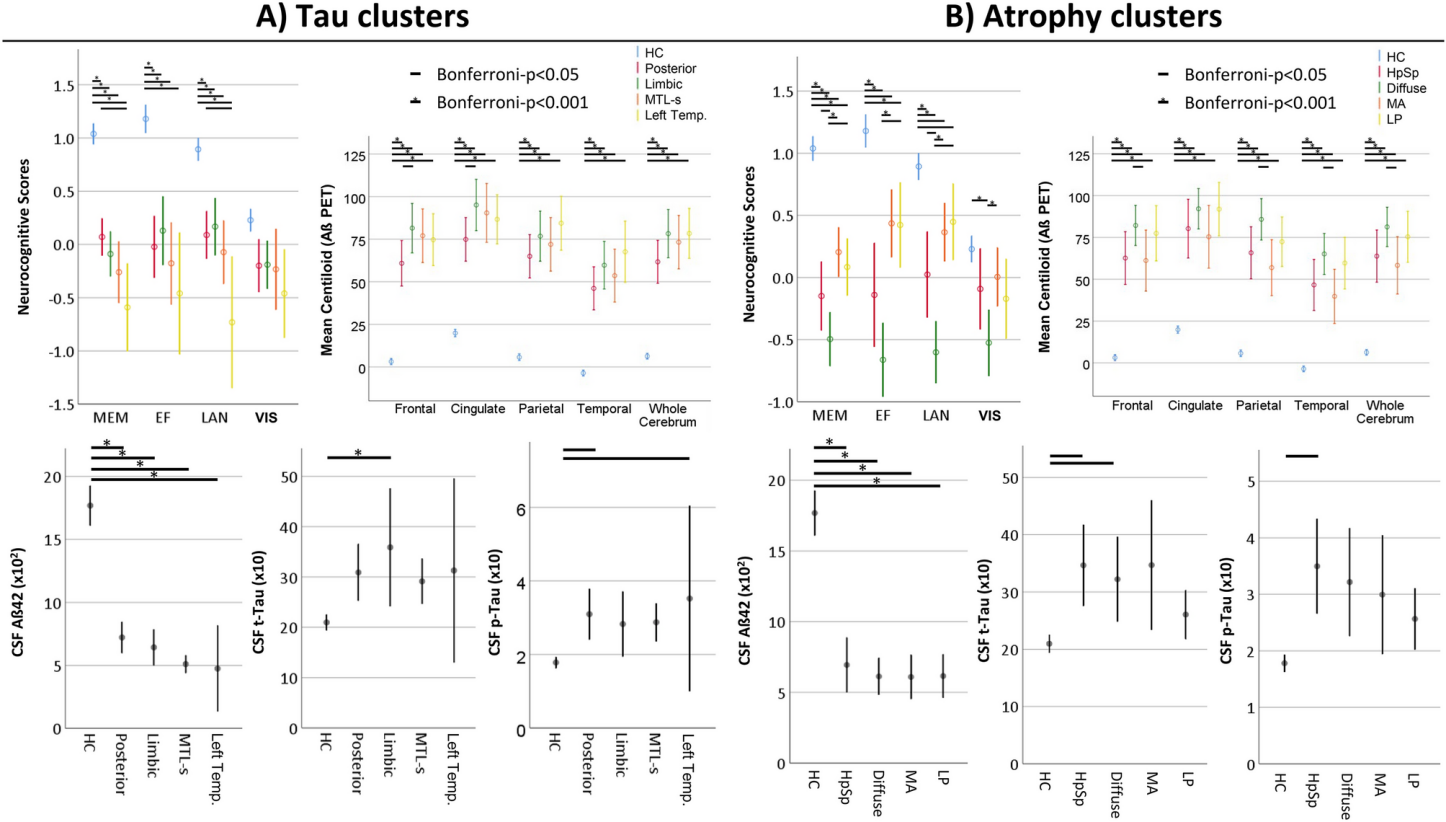
Baseline cognitive profile, CSF biomarker and Aβ PET centiloid level comparisons among tau (**A**) and atrophy (**B**) clusters. The vertical lines represent the standard errors. HC, healthy controls; MTL-s, medial temporal lobe sparing; Left Temp., left temporal; HpSp, hippocampal-sparing; MA, minimal atrophy; LP, limbic-predominant; MEM, memory; EF, executive function; LAN, language; VIS, visuospatial functioning; p-Tau, phosphorylated tau; t-Tau, total tau; Aβ42, amyloid β42; Aβ PET, amyloid-ß PET.

We examined the baseline characteristics of clusters of both imaging modalities by conducting cross-sectional group comparisons that are shown in Table 1 and Fig. 3. The tau PET clusters and atrophy clusters included more frequently *APOE* ε4 allele carriers compared to HC. We found that the MTL-sparing cluster was younger than posterior and limbic tau clusters. Regarding the clinical severity, posterior tau cluster revealed higher MMSE scores compared to left temporal tau cluster. Among atrophy clusters, only HpSp and diffuse atrophy clusters have significantly lower MMSE scores compared to HC, while diffuse atrophy cluster also differed from MA and LP clusters. Moreover, diffuse atrophy cluster revealed the highest CDR-global scores among the atrophy clusters. Importantly, clinical diagnosis did not differ among tau clusters, while diffuse atrophy cluster comprised more frequently Alzheimer’s disease dementia (ADD) diagnosis than MA and LP clusters. Differences in cognitive composite domain scores among tau PET clusters were observed in memory (MEM) and language (LAN), driven by higher scores in posterior tau cluster compared to left temporal tau cluster. The diffuse atrophy cluster revealed the lowest cognitive composite scores in all domains, while HpSp atrophy cluster did not differ from diffuse atrophy clusters in EF and VIS.

### Baseline biomarker comparisons of the tau PET and atrophy clusters

The baseline differences in non-imaging (Table 1 and Fig. 3) and imaging biomarkers (eTable 1 and Fig. 3) were examined. The CSF t-Tau concentrations in tau PET clusters were higher in limbic tau cluster at baseline compared to HC, while the CSF p-Tau concentrations were higher in posterior and left temporal tau PET clusters when compared to HC. When compared to HC, the HpSp atrophy cluster exhibited higher CSF t-Tau and p-Tau concentrations, while diffuse atrophy cluster revealed higher t-Tau concentrations. The CSF Aβ42 concentrations were lower and global and regional Aβ PET accumulation were higher in each tau and atrophy clusters compared to HC. The results point out to differences towards lower Aβ PET accumulation in the frontal lobe and the cingulate gyrus in the posterior tau PET subtype compared to limbic tau PET subtype as well as towards higher Aβ PET uptake in the summary composite region and subregions other than the cingulate region in the diffuse atrophy subtypes compared to MA subtype. When measured in composite region, each tau PET cluster had higher tau PET uptake values than HC, while atrophy clusters other than LP showed higher values from HC. The left temporal tau cluster had higher levels than posterior tau cluster and diffuse atrophy cluster showed the highest tau uptake in composite region among the atrophy clusters. Regarding the CTV:HV, each tau PET cluster and only diffuse and LP atrophy subtypes revealed higher ratio compared to HC. Moreover, the HpSp atrophy and MA clusters had lower CTV:HV levels than diffuse atrophy and both diffuse and LP atrophy clusters, respectively.

### Longitudinal characterization of tau PET and atrophy clusters

**Fig. 4:**
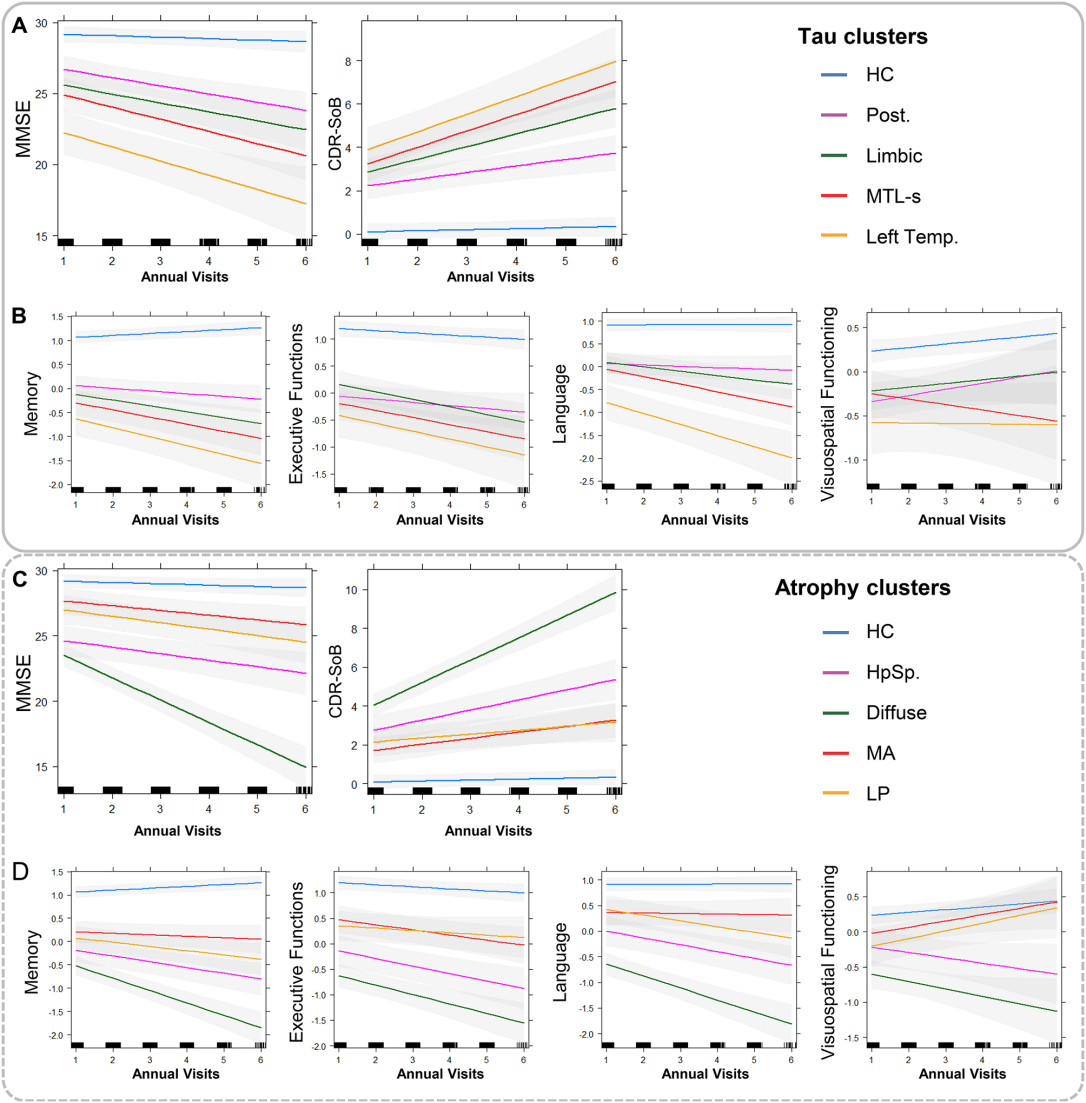
Longitudinal trajectories of clinical assessments (**A**) and cognitive assessments (**B**) in tau clusters and clinical assessments (**C**) and cognitive assessments (**D**) in atrophy clusters. *HC* Healthy controls; *Post* Posterior; *MTL-s* Medial temporal lobe sparing; *Left Temp.* Left temporal; *MEM* Memory; *EF* Executive function; *LAN* Language; *VIS* Visuospatial functioning; *CDR-SoB* Clinical dementia ratio-sum of boxes; *MMSE* Mini-mental state examination.

After the cross-sectional analyses, we investigated the longitudinal trajectories in tau PET and atrophy clusters. Among the tau PET clusters, the left temporal tau PET cluster exhibited the most rapid clinical progression trajectory followed by limbic and MTL-sparing tau clusters, particularly in terms of Clinical Dementia Rating—Sum of Boxes (CDR-SoB) scores (Table 2 and Fig. 4A), while all these three tau PET clusters showed significantly higher increase in CDR-SoB compared to posterior tau cluster (Table 3) in the post-hoc comparisons within ADS. Moreover, all tau PET clusters demonstrated significant decline decline in MEM (Table 2 and Fig. 4B), while the group comparison pointed out more pronounced decline in left temporal tau cluster compared to posterior and limbic tau clusters (Table 3). Interestingly, left temporal tau cluster exhibited the most rapid decline LAN compared to other tau PET clusters among ADS (Table 3 and Fig. 4B), while tau PET clusters except posterior revealed decline in LAN in the analysis including the entire cohort (Table 2). Among the atrophy clusters, only the diffuse atrophy subtype has revealed significant clinical decline, considering both MMSE and CDR-SoB (Table 2 and Fig. 4C), whereas post-hoc analyses revealed differences only with MA and LP clusters (Table 3). Also, the results of the group comparisons of annual change rates of MEM and LAN were very similar to clinical severity measures, revealing most rapid decline in diffuse atrophy cluster compared to MA and LP clusters (Table 3 and Fig. 4D). Moreover, HpSp atrophy cluster exhibited more pronounced decline compared to MA cluster in MEM at a marginal level of significance and LAN (Table 3 and Fig. 4D).

**Table 2:** Prediction of longitudinal trajectories in clinical severity and cognition over time in tau and atrophy clusters. Significant interactions were indicated in bold when Bonferroni-adjusted *p *< 0.05.

Dependent variable	Tau cluster				Atrophy clusters			
	Term interacting with time	β	SE	p-unc	Term interacting with time	β	SE	p-unc
MMSE	Posterior	− 0.11	0.13	0.38	HpSp	− 0.16	0.15	0.31
	Limbic	− 0.34	0.13	0.01	Diffuse	− **1.19**	**0.14**	** < 0.001**
	MTL-sparing	− 0.39	0.15	0.01	Minimal atrophy	− 0.03	0.13	0.81
	Left temporal	− **1.65**	**0.02**	** < 0.001**	Limbic-predominant	− 0.2	0.15	0.18
CDR-SoB	Posterior	− 0.01	0.06	0.88	HpSp	0.16	0.07	0.02
	Limbic	**0.19**	**0.06**	**0.001**	Diffuse	**0.37**	**0.06**	** < 0.001**
	MTL-sparing	**0.19**	**0.07**	**0.007**	Minimal atrophy	− 0.03	0.06	0.6
	Left temporal	**0.42**	**0.1**	** < 0.001**	Limbic-predominant	0.06	0.06	0.35
MEM	Posterior	− **0.2**	**0.06**	**0.002**	HpSp	− **0.29**	**0.07**	** < 0.001**
	Limbic	− **0.3**	**0.06**	** < 0.001**	Diffuse	− **0.57**	**0.07**	** < 0.001**
	MTL-sparing	− **0.35**	**0.07**	** < 0.001**	Minimal atrophy	− 0.14	0.06	0.03
	Left temporal	− **0.58**	**0.11**	** < 0.001**	Limbic-predominant	− **0.26**	**0.07**	** < 0.001**
EF	Posterior	− 0.02	0.06	0.76	HpSp	− 0.13	0.07	0.08
	Limbic	− 0.15	0.06	0.02	Diffuse	− 0.18	0.08	0.02
	MTL-sparing	− 0.14	0.07	0.07	Minimal atrophy	− 0.08	0.06	0.2
	Left temporal	− 0.08	0.11	0.5	Limbic-predominant	− 0.03	0.07	0.67
LAN	Posterior	− 0.04	0.07	0.58	HpSp	− **0.24**	**0.08**	**0.004**
	Limbic	− **0.25**	**0.07**	**0.001**	Diffuse	− **0.48**	**0.08**	** < 0.001**
	MTL-sparing	− **0.31**	**0.08**	** < 0.001**	Minimal atrophy	− 0.02	0.07	0.75
	Left temporal	− **0.53**	**0.12**	** < 0.001**	Limbic-predominant	− **0.22**	**0.08**	**0.008**
VIS	Posterior	0.02	0.1	0.83	HpSp	− 0.22	0.12	0.06
	Limbic	− 0.01	0.1	0.93	Diffuse	− **0.35**	**0.12**	**0.004**
	MTL-sparing	− 0.25	0.11	0.03	Minimal atrophy	0.06	0.1	0.53
	Left temporal	− 0.13	0.17	0.45	Limbic-predominant	0.11	0.11	0.34

*SE* Standard error; *unc* Uncorrected; *MMSE* Mini-mental-state examination; *CDR-SoB* Clinical Dementia Rating—Sum of Boxes; *MEM* Memory; *EF* Executive function; *LAN* Language; *VIS* Visuospatial functioning; *MTL* Medial temporal lobe; *HpSp* Hippocampal sparing.

**Table 3:** Differences in annual change rates of clinical severity (CDR-SoB) and cognition (cognitive composite domain scores) among tau and atrophy clusters. Significant results were indicated in bold when p-Bonferroni < 0.05. ^a^Set to zero because this parameter is redundant.

Tau PET clusters								
	Cluster	β	SE	*p*	p-Bonferroni			
					vs. Posterior	vs. Limbic	vs. MTL-sparing	vs. L Temp
CDR-SoB	Left temporal	**0.09**	**0.03**	**0.001**	**0.01**	1	1	–
	MTL-sparing	**0.06**	**0.02**	**0.01**	**0.04**	1	–	1
	Limbic	**0.07**	**0.02**	**0.002**	**0.01**	–	1	1
	Posterior	0^a^			–	**0.01**	**0.04**	**0.01**
MEM	Left temporal	** − 0.13**	**0.03**	**0.0001**	**0.0004**	**0.048**	0.38	–
	MTL-sparing	** − **0.06	0.03	0.02	0.1	1	–	0.38
	Limbic	** − **0.04	0.02	0.08	0.47	–	1	**0.048**
	Posterior	0^a^			–	0.47	0.1	**0.0004**
EF	Left temporal	** − **0.02	0.02	0.36	1	1	1	–
	MTL-sparing	** − **0.02	0.01	0.17	1	1	–	1
	Limbic	** − **0.02	0.01	0.08	0.48	–	1	1
	Posterior	0^a^			–	0.48	1	1
LAN	Left temporal	** − 0.15**	**0.03**	**0.000001**	**0.000004**	**0.004**	**0.004**	–
	MTL-sparing	** − **0.04	0.02	0.11	0.66	1	–	**0.004**
	Limbic	** − 0.04**	**0.02**	**0.04**	0.22	–	1	**0.004**
	Posterior	0^a^			–	0.22	0.66	**0.000004**
VIS	Left temporal	** − **0.02	0.01	0.18	1	1	1	–
	MTL-sparing	** − **0.02	0.01	0.11	0.65	1	–	1
	Limbic	** − **0.01	0.01	0.57	1	–	1	1
	Posterior	0^a^			–	1	0.65	1

Atrophy clusters								
	Cluster	β	SE	*p*	p-Bonferroni			
					vs. HpSp	vs. Diffuse	vs. Min. atrophy	vs. LP
CDR-SoB	Limbic-predominant	** − **0.04	0.03	0.11	0.67	0.01	1	–
	Minimal atrophy	** − 0.06**	**0.02**	**0.01**	0.08	**0.0002**	–	1
	Diffuse	**0.03**	**0.02**	**0.14**	0.84	–	**0.0002**	**0.01**
	HpSp	0^a^			–	0.84	0.08	0.67
MEM	Limbic-predominant	0.02	0.03	0.4	1	0.02	0.51	–
	Minimal atrophy	**0.07**	**0.03**	**0.01**	0.06	**0.000005**	–	0.51
	Diffuse	** − 0.05**	**0.03**	**0.04**	0.21	–	**0.000005**	**0.02**
	HpSp	0^a^			–	0.21	0.06	1
EF	Limbic-predominant	0.02	0.02	0.24	1	1	1	–
	Minimal atrophy	0.01	0.01	0.38	1	1	–	1
	Diffuse	** − **0.0002	0.01	0.99	1	–	1	1
	HpSp	0^a^			–	1	1	1
LAN	Limbic-predominant	0.04	0.03	0.13	0.78	0.0005	1	–
	Minimal atrophy	**0.07**	**0.03**	**0.01**	**0.046**	**0.000001**	–	1
	Diffuse	** − 0.06**	**0.02**	**0.02**	0.11	–	**0.000001**	**0.0005**
	HpSp	0^a^			–	0.11	**0.046**	0.78
VIS	Limbic-predominant	0.03	0.01	0.02	0.11	0.36	1	–
	Minimal atrophy	0.03	0.01	0.02	0.11	0.41	–	1
	Diffuse	0.01	0.01	0.47	1	–	0.41	0.36
	HpSp	0^a^			–	1	0.11	0.11

*SE* Standard error; *CDR-SoB* Clinical dementia rating—sum of boxes; *MEM* Memory; *EF* Executive function; *LAN* Language; *VIS* Visuospatial functioning; *MTL* Medial temporal lobe; *L. Temp.* Left temporal; *HpSp* Hippocampal sparing; Min. atrophy; *LP* Limbic-predominant.

**Fig. 5:**
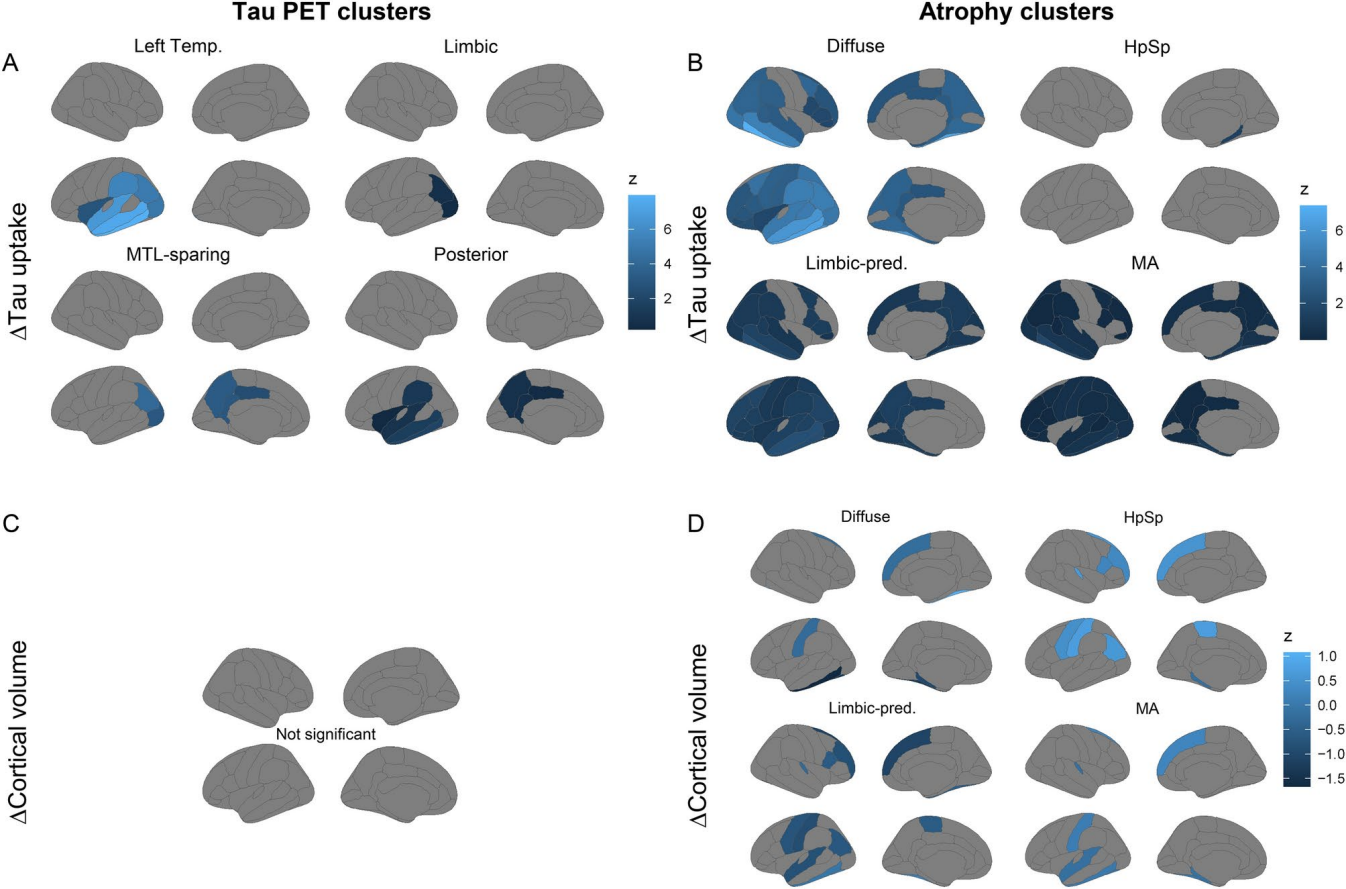
Annual change rates (in z-scores) of tau PET and cortical volumes are shown as group means for tau PET clusters (**A** and **C**) and atrophy clusters (**B** and **D**). The Desikan-Killiany atlas regions are shown, including only regions of interest with significant differences in pairwise comparisons (overall-p-FDR < 0.05 and pairwise-p-Bonferroni < 0.05). Left Temp, left temporal; MTL-sparing, medial temporal lobe sparing; HpSp, hippocampal sparing; Limbic-pred., limbic-predominant.

More, we compared the progression in tau PET uptake and atrophy among tau PET and atrophy clusters over time. Here, we tested differences in annual change rates of each modality based on regions of interest (ROIs) by using Desikan-Killiany atlas. We observed that the tau accumulation particularly but not limited to regions exhibiting initial high tau binding at baseline both among tau PET and atrophy clusters (Fig. 5A-B, eFigure 5A,B and eTable 2).

The left temporal tau PET cluster revealed more rapid tau accumulation in left parietal and occipital regions (inferior parietal and lateral occipital) compared to the limbic tau cluster and in left temporal, parietal and insular regions (inferior temporal, insula, middle temporal, superior temporal, supramarginal, and temporal pole) compared to the posterior tau cluster. Moreover, the MTL-sparing tau PET cluster showed a faster increase in tau uptake in left inferior parietal and lateral occipital cortices compared to the limbic tau cluster and in medial regions (isthmus cingulate, posterior cingulate, and precuneus) of left hemisphere when compared to the posterior tau cluster. Comparing the tau longitudinal PET uptake among atrophy clusters, we found mainly more rapid increase in diffuse atrophy cluster than MA and LP clusters in a widespread fashion, while the differences in the left frontal pole and left insular cortex (diffuse > LP) as well as differences in the right entorhinal cortex, pars triangularis, and right rostral middle frontal cortex (diffuse > MA). Our findings also highlight a slower increase in tau uptake in the HpSp atrophy cluster compared to the diffuse atrophy cluster, particularly in the left temporal pole and the right parahippocampus.

Regarding the annual change rates of cortical volumes, tau PET clusters revealed no differences (eTable 3 and Fig. 5C), while atrophy clusters exhibited notable differences (Fig. 5D, eFigure 5D and eTable 3). Specifically, the diffuse atrophy clusters exhibited the fastest volume loss in the temporal and frontal regions compared to the HpSp and MA clusters, with the LP atrophy cluster falling in between. An exception to this pattern was observed in the right fusiform gyrus, where the LP atrophy cluster displayed a faster volume loss compared to the diffuse atrophy cluster.

## Discussion

Advanced image acquisition and analysis can uncover heterogeneity in neurological diseases, previously viewed as homogenous entities due to limited assessment methods. In ADS, imaging-based clusters have been proposed recently, suggesting that the assumption of a uniform tau spreading pattern does not appropriately consider the variability between patients^22^. Similar heterogeneity has been shown for AD atrophy patterns^6,21,24^, challenging the presumption of a “typical AD” biological phenotype.

In the present study, we performed for the first time a head-to-head comparison of the cross-sectional overlap and disease progression among clusters derived from tau PET and structural MRI within the same subjects. We identified distinct clusters of tau PET binding and cortical atrophy patterns in ADS that were also accompanied by distinguishable characteristics in non-imaging biomarkers and clinical and neuropsychological assessments. Tau and atrophy clusters showed similarities between cortical atrophy and tau binding patterns at baseline, while the subjects’ allocation showed considerable heterogeneity between modalities. Moreover, the longitudinal analyses revealed substantial heterogeneity in the progression of imaging biomarkers among clusters in both modalities. Regarding the possible influence of disease burden on the clustering as shown in Table 1, the tau PET clusters were similar in clinical diagnosis frequency of mild cognitive impairment (MCI) or ADD at baseline. Conversely, the diffuse atrophy cluster had significantly higher rates of dementia diagnosis compared to MA and LP atrophy clusters.

In line with recent research suggesting at least four distinct tau spreading patterns^22^, we identify four tau PET clusters in ADS using an unsupervised similarity-based imaging clustering approach. It has been proposed that tau and atrophy patterns can be characterized along the severity or the typicality axes^6^. Characteristic differences in progression rate and spatial pattern of each cluster and for both imaging biomarkers support the notion of independent biologically defined entities.

The tau and atrophy clusters revealed significant correlations between cortical tau PET binding and atrophy levels on vertex level in each cluster, while tau clusters showed more distinct patterns than atrophy clusters. With this, we observed that left temporal tau PET and LP atrophy clusters showed low spatial correlation between both modalities. The latter observation is in line with a previous study on typical and atypical AD phenotypes, showing that greater asymmetry can be related to lower correlation between tau binding and cortical thickness^28^. A recent study suggested that an association between high typicality in atrophy pattern and less frequent comorbidities in post-mortem examination, also underlining possible impacts of non–AD pathological changes on radiologic findings^23^. Our findings also indicate that clinical differences at baseline point towards distinct neuropsychological test profiles in different tau clusters, while atrophy clusters differed in cognitive impairment to a lesser extent. Given these differences, the tau and atrophy clusters unsurprisingly showed limited overlap, suggesting that the heterogeneity in tau PET and structural MRI patterns might represent substantially different disease processes such as differential roles of comorbid non–AD pathological changes, i.e., aging, vascular pathologies, α-synuclein, and TDP-43^25,29^. However, the tau uptake may show stronger correlations with cortical volume loss in later disease stages, given the chronological sequence of AD biomarker in disease progression^30^, which might be supported by the findings in post-mortem studies^28^. Importantly, cortical atrophy can be understood as a downstream event that is partially related to tau pathology^4,25,31^, suggesting a temporal relationship between tau and atrophy spreading patterns that can have an impact on the derived clusters within both modalities. However, the heterogeneity in their temporal and spatial relationship^32^ that is likely to be affected by factors such as disease severity^31^, demographics and white matter hyperintensity volumes^33^, is not yet entirely understood. Of note, due to our focus on the characterization of clustered groups, we assessed the longitudinal change from the perspective of comparisons among respective clusters and not as spreading patterns differing from HC participants. Therefore, no spreading patterns should be concluded in isolation within a cluster from the analyses, but rather notable group differences as possible preference in spreading patterns in particular cortical regions. Also, considering the effects of regional tau pathology on the decline in global cognition, a recent study suggested that the relationship between regional tau PET uptake and cognition might be partly mediated by atrophy in the suggested Aβ-tau-atrophy pathway, which is slightly increasing in ADD compared to MCI^25^.

Considering the difference in group comparisons, Aβ PET binding in frontal and cingulate regions differed in the limbic tau PET cluster compared to the posterior tau PET cluster, while only atrophy clusters revealed a difference global Aβ PET uptake in favor of higher uptake diffuse atrophy compared to MA cluster. However, studies have reported inconsistent Aβ burden among AD subtypes, with only a few studies showing no difference in neuropathologically derived global Aβ burden^13^ and global Aβ PET binding among atrophy subtypes^34^ and one study reporting higher global Aβ PET binding in temporal and parietal regions in LP than in typical AD subtype^28^. The difference between the results might originate from differences in the included diagnostic groups (ADD vs. ADS), as Aβ pathology occurs already in the preclinical stages of the disease and approaches a plateau in clinical AD^35^.

Another important finding of our study was the distinguishable profiles and trajectories of cognitive impairment among both tau and atrophy clusters, supporting the neuropathological heterogeneity in AD^10^, in line with the previous multimodal imaging studies^36^ and neuropathological analyses^37^ on atypical AD. The atrophy clusters, in contrast to tau clusters, revealed no cluster with pronounced occipital atrophy, while both the HpSp and diffuse atrophy clusters revealed atrophy in occipital areas, in line with a recent meta-analysis^6^. Moreover, the left temporal tau PET cluster revealed imaging characteristics comparable to some extent to the logopenic-variant of AD, also characterized by relatively impaired LAN at baseline and left hemisphere-lateralized temporal tau pathology as well as a rapid cognitive decline. This clinical phenotype was suggested as an AD pathology-associated form of primary progressive aphasia^10^. In contrast to tau clusters, atrophy clusters revealed no cluster with a marked language deficit or asymmetrical atrophy pattern. Also, the observed clusters might present distinguishable involvement patterns of cognitive neural networks, so that the different cognitive trajectories are affected preferentially, as presented in differences longitudinal analyses of cognitive domain scores, especially among tau PET clusters. Moreover, in terms of demographics, the MTL-sparing tau cluster was younger than other tau clusters. This finding is in line with the previous finding demonstrating that lower age was associated with a higher rate of tau positivity in the Braak V-VI ROI in Aβ-positive MCI^38^.

It has been previously suggested that the biological foundation of heterogeneity in AD arises from individual differences in the initial seeding sites or heterogeneity of the tau molecule^39,40^. Furthermore, only it has become clear recently that tau pathology is strongly associated with resting state functional connectivity^40^, the interaction with network connectivity may therefore play an important role in the regional variance of tau PET uptake. Another explanation might also be related to differences in cognitive reserve or brain reserve as a factor of resilience against expansion of tau pathology and variability in disease spreading patterns^6,33,39,41^. A further important concept is brain maintenance that some individuals are more likely to preserve the brain morphology over the course of AD possibly due to processes like neurogenesis and more effective clearance of pathological changes^42^. Of note, higher levels of 18F-flortaucipir (AV-1451) tau PET that reflects filamentous tau has also been reported in the medial and inferior temporal lobe in healthy elderly^43,44^. We included age as covariate in our analyses that might have neutralized aging-related heterogeneity which should be explored in further studies. Moreover, remarkable increase in wide-spread cerebral tau deposition has also been found in patients with vascular cognitive impairment without concomitant AD^43,45^. Future studies are needed to explore the impact of concomitant cerebrovascular disease on the tau heterogeneity in AD.

Our results are strongly supported by the remarkably similar results of a previous study that included a high number of patients from four independent cohorts^22^. Another strength of our study is the head-to-head comparison of multimodal approaches in clustering patients in ADS. A few limitations must be acknowledged in the present analysis. Aβ PET was used as an inclusion criterion if CSF markers were not available. This may have introduced some heterogeneity, but both biomarkers were suggested to have equally high diagnostic accuracy for binary classification^46^. Some further diagnostic misclassifications may have been introduced since no histopathological verification is available in ADNI, but the adopted biomarker-based classification approach allows for a reasonably good approximation to neuropathological assessments. Further limitations are a non-diverse participant profile in ADNI study, which limits the possible further variability in pathological or clinical manifestations, and moderate follow-up duration, although one of the biggest and most comprehensive AD datasets available worldwide was used for this analysis. However, the available cases with follow-up data, especially missing longitudinal tau PET data in left temporal tau cluster presents a considerable limitation in the related longitudinal analyses. A possible limitation of the unbiased clustering approach used in this study as with all unsupervised clustering methods is cluster allocation of individuals and its dependence on different pre-adjustments in the clustering algorithms. We compared the characters of derived clusters with the above-mentioned previous studies that included various cohorts and various clustering methods, resulting in comparable clusters in AD. However, further analyses are needed to validate (e.g. by using direct comparisons) our clustering approach with other data-driven approaches such as Subtype and Stage Inference (SuStaIn)^47^ in the same patient cohorts and test the stability/replicability of observed clusters by including independent cohorts. Further evidence is needed to gain a better understanding of these methodological limitations. Our results suggest that studies should address possible endophenotypes of AD while testing diagnostic methods and biological staging, e.g., binarization of pathological changes. Future studies should also consider possible overlaps between clinical phenotypes of AD and biological endophenotypes and utilize additional modalities, i.e., anatomical and functional connectivity. Moreover, combinative approaches to differentiate covariance patterns of multiple modalities (i.e., PET and MRI) can be examined to address converging contributions of each modality.

The presented results foster our understanding of individual differences in imaging characteristics of distinct clusters across the ADS, yielding important insights for individual disease progress prediction and precision medicine approaches. For new therapies targeting tau^48,49^, or for tau as a downstream marker in anti-Aβ therapy^50^, targeting patients within a certain tau PET or atrophy-based pattern could be an approach that improves effectiveness and safety. Furthermore, the optimal timing of treatment initiation could depend on the underlying cluster pattern, as tau positivity in conventional estimation methods may be affected by distinguishable spreading patterns especially in preclinical AD.

## Materials and methods

Data included in this study originate from the AD Neuroimaging Initiative (ADNI) launched in October 2004 (ClinicalTrials.gov IDs: NCT02854033, NCT01231971). As per the ADNI protocol, all procedures performed in the study involving human participants were in accordance with the ethical standards of the institutional and/or national research committees. Experiments were undertaken with the understanding and written consent of each subject. All local institutional review boards and ethical committees approved the study protocol.

### Study participants

Baseline data was accessed in July 2020 and longitudinal data were added in March 2023. We included subjects from ADNI who received T1 structural brain MRI and tau PET (^18^F-AV1451 PET) (N = 657). N = 45 did not pass the quality assessment or failed in the FreeSurfer analysis resulting in N = 612. Only participants with available Aβ PET (^18^F-AV45 PET or ^18^F-FBB PET) and/or CSF results and neuropsychological testing (N = 478) were considered for this analysis. After excluding Aβ positive clinically unimpaired participants (N = 154) and participants with suspected non-AD pathology (Aβ negative but tau PET positive) (N = 4), the final study cohort resulted in N = 154 Aβ negative and cognitively normal healthy controls (HC, 74 years, 85 female) and N = 166 Aβ positive MCIADD patients along the ADS) (75 years, 76 female). We defined Aβ positivity by using a cut-off of 0.075 for the CSF Aβ42/40 ratio^51^; if Aβ CSF results were not available, Aβ PET was used instead with cut-offs for global normalized whole cerebrum standardized uptake value ratio of (SUVr)_AV45_ > 1.11^52,53^ and SUVr_FBB_ > 1.08 (defined in the Florbetaben (FBB) processing methods, http://adni.loni.usc.edu/, accessed at 26/08/2021). The Aβ CSF analysis and Aβ PET scans that were used to determine Aβ positivity were acquired in a timeframe ± 180 days prior to/after the Tau-PET scan (mean time between CSF Aβ and tau-PET = 6 ± 38 days; mean time between Aβ PET and tau-PET = 25 ± 38 days). MCI and AD diagnoses were determined using standardized criteria, reported in the ADNI3 Protocol, Protocol Number: ATRI-001 (http://adni.loni.usc.edu/wp-content/themes/freshnews-dev-v2/documents/clinical/ADNI3_Protocol.pdf).

### MRI acquisition and processing

The ADNI MRI acquisition protocol is reported elsewhere (http://adni.loni.usc.edu/methods/mri-tool/mri-acquisition/). All whole-brain T1-Magnetization Prepared—Rapid Gradient Echo (MPRAGE) or inversion pulse spoiled gradient recalled (IR-SPGR) T1-weighted images (Slice thickness 1–1.2 mm; TR, 2300–3000 ms; TE 2.9- 3.5 ms; FoV, 256 × 256 cm^2^) were processed in FreeSurfer (v6, http://surfer.nmr.mgh.harvard.edu/). Segmentation and parcellation were performed using the recon-all pipeline, including registration to standard space, intensity normalization, brain extraction, tissue type classification, surface reconstruction and probabilistic anatomical labeling^54^. The recon-all results were visually inspected for accuracy and corrected as needed. Cortical and hippocampal volumes were derived from segmentations using the FreeSurfer (Desikan-Killiany) atlas. The longitudinal analyses of cortical volume values in Desikan-Killiany atlas regions were conducted by including the data processed as reported elsewhere^55^. The CTV:HV was estimated by dividing the bilateral total hippocampal volumes^13^ by the bilateral total cortical volume using all cortical FreeSurfer (Desikan-Killiany) atlas regions.

### PET acquisition and processing

18F-florbetapir (AV45) and FBB Aβ PET and AV-1451 tau PET were acquired using the following parameters: AV45, 370 MBq (10.0 mCi) ± 10%, 20 min (4 × 5 min frames) acquisition at 50–70 min post-injection; FBB, 300 MBq (8.1 mCi) ± 10%, 20 min (4 × 5 min frames) acquisition at 90–110 min post-injection; AV-1451, 370 MBq (10.0 mCi) ± 10%, 30 min (6 × 5 min frames) acquisition at 75–105 min post-injection (details available elsewhere, http://adni.loni.usc.edu/). Cortical tau depositions were assessed by 18F-AV1451 imaging using the PETSurfer tool in FreeSurfer^56,57^ to derive fsaverage surface to perform surface-based analysis including clustering and regional group comparisons among HC and clusters at baseline. For the longitudinal analyses we included the mean SUVr in the Desikan-Killiany atlas regions, as described elsewhere^55^.

We selected PET scans with an available anatomical MRI within ± 180 days from the date of the corresponding PET for baseline analyses. All 18F-AV1451 scans were downloaded in the most fully preprocessed format available on LONI (https://ida.loni.usc.edu/; series description: AV1451 Coreg, Avg, Std Img and Vox Siz, Uniform Resolution) and were co-registered to the corresponding anatomical MRI. Partial volume correction was performed using the previously created high-resolution segmentation of the anatomical MRI using the Muller-Gartner method^58^. The cerebellar cortex was used as the reference region for intensity scaling. The results were sampled onto the FreeSurfer fsaverage surface to perform surface-based analysis halfway between the white matter and pial surface via the individual surface. The results were spatially smoothed before vertex-wise comparisons on the surface using a 5 mm FWHM Gaussian kernel.

The cut-point distinguishing between ADS and HC was defined at a tau PET meta ROI SUVr value of 1.37, as reported elsewhere for ADNI cohort^59^. The tau PET meta ROI included the mean uptake of voxels in the entorhinal cortex, amygdala, fusiform, inferior temporal, and middle-temporal ROIs^60^. The baseline cut-point for tau pathology positivity was conducted using summary results from LONI (http://adni.loni.usc.edu/, accessed at 05/03/2023), using standardized SUVr for inferior cerebellar gray matter.

The preprocessed summary data were used for assessing whole brain SUVR values of AV45 and FBB Aβ PET (http://adni.loni.usc.edu/, accessed at 05/03/2023). An Aβ PET meta ROI was used to calculate an Aβ PET composite score as mean florbetapir uptake within all cortical regions comprising the four large brain regions (frontal, anterior/posterior cingulate, lateral parietal, lateral temporal) as shown previously^61^. For ^18^F-FBB and ^18^F-AV45 PET SUVr calculations, the whole cerebellum was used as a reference region, as described elsewhere^53^, and no partial volume correction was applied. Of note, to obtain comparable quantification of the Aβ burden across tracers, we used the following formula for centiloid calculation as recommended for the ADNI pipeline: AV45 centiloid = 196.9 × SUVr_FBP_—196.03, and FBB centiloid = 159.08 × SUVr_FBB_—151.65.

### Clinical and cognitive characteristics and CSF biomarkers

Participants underwent extensive neuropsychological testing to assess performance across cognitive domains, enabling the calculation of cognitive composite domain scores of the following domains: MEM, executive functions (EF), LAN, and visual-spatial functions (VIS)^62^. Examination of neuropsychological testing, which was within a timeframe of ± 180 days before/after the Tau-PET date (mean time between neuropsychology visit and Tau-PET = 14 ± 41 days). The overall severity of dementia was quantified using the CDR-SoB score. Additionally, the MMSE score was used, given its high relevance in everyday clinical practice. CSF biomarkers were assessed using established commercially available analysis kits, following standardized procedures. The CSF concentrations of Aβ40, Aβ42, t-Tau and p-tau181 were quantified in aliquoted samples using the electrochemiluminescence immunoassay Elecsys on a fully automated Elecsys cobas e 601 instrument (Roche Diagnostics GmbH, Penzberg, Germany) using a single lot of reagent for each of the four measured biomarkers.

### Identification of clusters in tau-PET and MRI

To identify biological clusters, an unbiased and data-driven clustering approach based on pattern similarity, the Louvain algorithm^19,63^ using the consensus method^24,64^, was applied to both the tau PET and MRI datasets separately in the same patient cohort. Therefore, every patient in ADS group was assigned for one tau PET and atrophy clusters.

Individual vertex-wise cortical SUVr values for tau PET and cortical thickness values for MRI datasets obtained from each participant using PETSurfer and FreeSurfer, respectively, were registered and resampled to the FreeSurfer standard subject template (fsaverage6), with 40,962 vertices for each hemisphere^19,24^. Subsequent analyses were performed using in-house MATLAB (The MathWorks, Inc.) scripts when not otherwise mentioned.

Z-scores of both the vertex-wise tau PET tracer uptake and the cortical thickness in the Alzheimer’s disease spectrum (ADS) group were calculated by vertex-wise subtraction of the ADS group’s tau SUVr/thickness from the controls’ tau SUVr/thickness divided by the standard deviation. For clarity, we computed vertex-wise z-scores of tau uptake for each subject in ADS with respect to the distribution in the HC (computed as z-score_vertex_ = (SUVr_vertex_^ADsubject^ − x̄SUVr_vertex_^HC^)/σSUVr_vertex_^HC^, in accordance with the previous work of Park et al.^19^, while z-scores of atrophy levels were computed by replacing SUVr with cortical thickness in this formula. The resulting z-score vectors were consecutively concatenated, and a similarity matrix of correlation coefficients between the obtained z-score vectors of any two subjects in the ADS group was calculated.

To identify tau and atrophy clusters of AD, an unbiased and data-driven cluster detection approach using the Louvain community analysis method implemented in the brain connectivity toolbox^63^, modified using the consensus clustering approach^24,64^ was applied. This clustering approach showed fairly high reproducibility and remarkable inter-dataset consistency using cortical thickness data^19^, which is also suggested as suitable for high-dimensional data^65^. Moreover, we applied the consensus clustering approach to obtain stable results through 1,000 iterations with a correction of individual-level modular decomposition^64^. We used a gamma value of 1.2 for tau clusters and 1.32 for atrophy clusters. The gamma value is a resolution parameter of the Louvain community structure analysis controlling the number of clusters and consecutively the level of clustering, with a smaller value resulting in a smaller number of clusters^65^. The gamma value was controlled to obtain a four-cluster solution and clusters comparable to previous tau PET^66^ and atrophy^21^ clustering patterns by using the smallest possible gamma value for the given number of clusters, as reported previously^19,24^.

To evaluate the stability of these clusters, we applied Leave-One-Out (LOO) cross-validation. This method randomly left out a small number of patients n ∈ {1:33} from the ADS group, recalculated the correlation matrix with the remaining data, and then reapplied the Louvain-based consensus clustering approach to the reduced dataset. The partition of each cluster in the clustering of the entire ADS group (n = 166, as for the main analyses of this study) resulted in what we termed the Consensus Initial Partition (CIP), serving as a reference point for comparison. Of note, the gamma value remained unchanged between CIP and any iterations. To quantify the similarity between the reference CIP clustering solution and each LOO iteration, we employed the Rand Index (RI)^67^. This metric assesses clustering agreement by examining the proportion of sample pairs that remain in the same or different clusters across the two solutions and allows to test the robustness of the observed clusters. Consequently, each LOO clustering assignment was compared against the CIP. We demonstrated that RI values were comparable and fluctuated ~ 0.6 (median value) for tau clusters and ~ 0.71 (median value) for atrophy clusters, across different LOO sample sizes, underlining the robustness of the identified clusters (eFigure 6). Of note, 10 repetitions of the same procedure confirmed the stability of the clusters, revealing median values of RI between 0.599–0.602 for tau and 0.706–0.715.

### Statistical analysis

SPSS (IBM, v26), R (https://www.r-project.org/, RStudio 2021.09.1) and MatLab (2017b) were used for statistical analyses. Graphics were generated using SPSS or ggplot2 package (https://ggplot2.tidyverse.org/articles/ggplot2-in-packages.html) in R and FreeView and ggseg package in R (https://ggseg.github.io/ggseg/) for brain visualizations.

Cluster group differences in relevant confounding variables (age, sex, binarized *APOE ε4* genotype and educational years) were compared with Kruskal–Wallis tests for continuous variables and chi-square tests for binary variables, as appropriate. All imaging and CSF biomarker analyses and cognitive and clinical assessments were also adjusted to account for different study sites. All analyses of MRI measures (hippocampal volumes and CTV:HV) were adjusted additionally for estimated total intracranial volumes. CSF biomarker scores, neurocognitive assessments and cognitive composite domain scores, SUVr of tau PET and Aβ PET, and MRI-derived hippocampal volumes were compared in the entire cohort and between clusters using an Analysis of Covariance (ANCOVA), adjusted for socio-demographical (age, sex, and educational years) and genetic confounders (binarized *APOE ε4* genotype) and study sites, when applicable. Post-hoc pairwise comparisons were Bonferroni corrected as appropriate. Results were considered significant at *p* < 0.05 (two-tailed).

The longitudinal changes in clinical and biological disease markers were compared among clusters (separately for tau and atrophy clusters) using linear mixed models (lmer in lme4-package), including time and intercept as a random factor, adjusted for age, sex, *APOE ε4* carrier status, years of education and study sites, as well as clinical diagnosis. The restricted maximum likelihood criterion approach was used. Bonferroni correction for multiple testing was applied, and results were considered when α < 0.05. Of note, we transformed MMSE by using logarithmic transformation (i.e., each variable is replaced with its logarithm using base 10) and CDR-SoB by using square root transformation (i.e. each variable is replaced with its square root) due to skewness in the data distribution. The available longitudinal data of clinical assessments and cognitive composite scores in each timepoint are shown in eTable 4. Moreover, we calculated annual change rates of CDR-SoB and single cognitive domain scores as slopes by linear mixed models adjusted for study sites and including time as a random factor. Consequently, we tested the group differences in annual change rates among clusters by using general linear models in tau and atrophy clusters separately, adjusted for age, sex, educational years, *APOE ε4* carrier status and clinical diagnosis (MCI or ADD). The Bonferroni correction was applied for pairwise comparisons and results were considered significant when p-Bonferroni < 0.05.

Average z-scores were calculated using age and sex-matched HC of tau uptake/cortical thickness for each tau cluster. Statistical differences in tau and atrophy levels between z-score maps of each cluster and all remaining participants in ADS were analyzed. Furthermore, each cluster z-map was compared to each other cluster using cortical z-score maps registered to the FreeSurfer standard subject template (fsaverage) by using two-tailed, two-sample unpaired n = 1000 permutation-based *t*-tests in FSL-PALM (Permutation Analysis of Linear Models)^68^, applying Threshold Free Cluster Enhancement (TFCE) and controlling for family-wise error rate (FWE). Results were considered significant when FWE *p* < 0.05. The most and least atrophy regions were defined by comparing any cluster with the rest of the patients in the ADS group. The covariance of atrophy levels and tau PET uptake between atrophy and tau clusters was tested as ROI-level correlations using z-scores of cortical volumes (for atrophy clusters) and tau PET SUVr (for tau clusters). The Spearman rank correlations were tested between clusters’ z-scores (cortical regions of Desikan-Killiany atlas) of one modality versus the z-scores across all ROIs of other modality. Since the higher values of tau PET and lower cortical volume values indicate a more advanced pathology, we corrected the resulting inverse associations by multiplying cortical volume by − 1 before calculating the rank correlations. Moreover, we compared cluster allocations also by applying chi-square test among the tau PET and atrophy clusters.

Longitudinal tau PET and cortical volumes for clusters were analyzed using adjusted annual change rates for each atlas region in Desikan-Killiany atlas, which were standardized over values of HC (z-scores). In the participants with at least one follow-up visit, the annual change rates were derived as slopes by linear mixed models that included time and intercept as random factors, adjusted for age, sex and *APOE ε4* carrier status. Z-scores of annual change rates were then compared among clusters of both modalities using non-parametric Kruskal–Wallis tests. The overall p values were corrected for Atlas regions (cortical regions, N = 34) using the false discovery rate^69^. The ggseg R-package^70^ was used to visualize each group’s mean annual change rates of tau PET and cortical volumes. Only the participants with at least two image data were included in the longitudinal imaging analysis, so the number of available data is shown in eFigure 4. The follow-up data (same for MRI and tau PET) showed a median follow-up duration of 1.85 years (minimum 0.58 and maximum 5.31), and the duration did not differ among the clusters (among tau PET clusters *p* = 0.84 and atrophy clusters *p* = 0.22) when compared by using Kruskal–Wallis tests).

Additionally, we showed the most- and least-affected regions in each modality to visualize contrasts between subgroups. Therefore, all subjects within a given cluster were compared with all remaining participants within this modality. Furthermore, we performed a vertex-wise regression analysis between tau PET and cortical atrophy z-scores using PALM to assess the spatial correlation between both entities.

## Supplementary Information

Supplementary Information.

## Data Availability

All ADNI data are deposited in a publicly accessible repository, which can be accessed at http://adni.loni.usc.edu. Correspondence regarding the study should be addressed to Boris-Stephan Rauchmann.

## Abbreviations

AD: Alzheimer’s disease
ADS: Alzheimer’s disease spectrum
ADD: Alzheimer’s disease dementia
ADNI: Alzheimer’s disease neuroimaging initiative
aRI: Adjusted rand index
AV-1451: 18F-flortaucipir
AV45: 18F-florbetapir
Aβ: Amyloid-β42
*ApoE*: Apolipoprotein E
CDR: Clinical dementia rating
CDR-SoB: Clinical dementia rating sum of boxes
CSF: Cerebrospinal fluid
CTV HV: The ratio of cortical volume to hippocampal volume
EF: Composite domain score for executive functions
FBB: Florbetaben
HC: Healthy control
HpSp: Hippocampal-sparing
MA: Minimal atrophy
MCI: Mild cognitive impairment
LAN: Composite domain score for language
LP: Limbic-predominant
MEM: Composite domain score for memory
MMSE: Mini mental state examination
MTL-sparing: Medial temporal lobe-sparing
MRI: Magnetic resonance imaging
PET: Positron emission tomography
ROI: Region of interest
p-Tau: Phosphorylated tau 181
SUVr: Standardized uptake value ratio
t-Tau: Total tau
VIS: Composite domain score for visual-spatial functions
