# Supplementary material for: Multimodal and longitudinal characterization of distinct tau and atrophy clusters in Alzheimer’s disease spectrum
Source: Sci Rep. 2025 May 25;15:18142. doi: 10.1038/s41598-025-98338-9 (PMC12104337; doi:10.1038/s41598-025-98338-9)
Supplement: Supplementary file 1 — Supplementary Information. [file 41598_2025_98338_MOESM1_ESM.docx]

**eFigure 1.** Comparison of z-scores of tau uptake among tau clusters with FWE-corrected p (A) and uncorrected (B). Comparison of z-scores of atrophy levels in atrophy clusters (C).

Abbreviations: Post., posterior; MTL-s, medial temporal lobe sparing; Left T., left temporal; HpSp, hippocampal-sparing; MA, minimal atrophy; LP, limbic-predominant; FWE, family-wise error; unc, uncorrected; n.s., not significant.


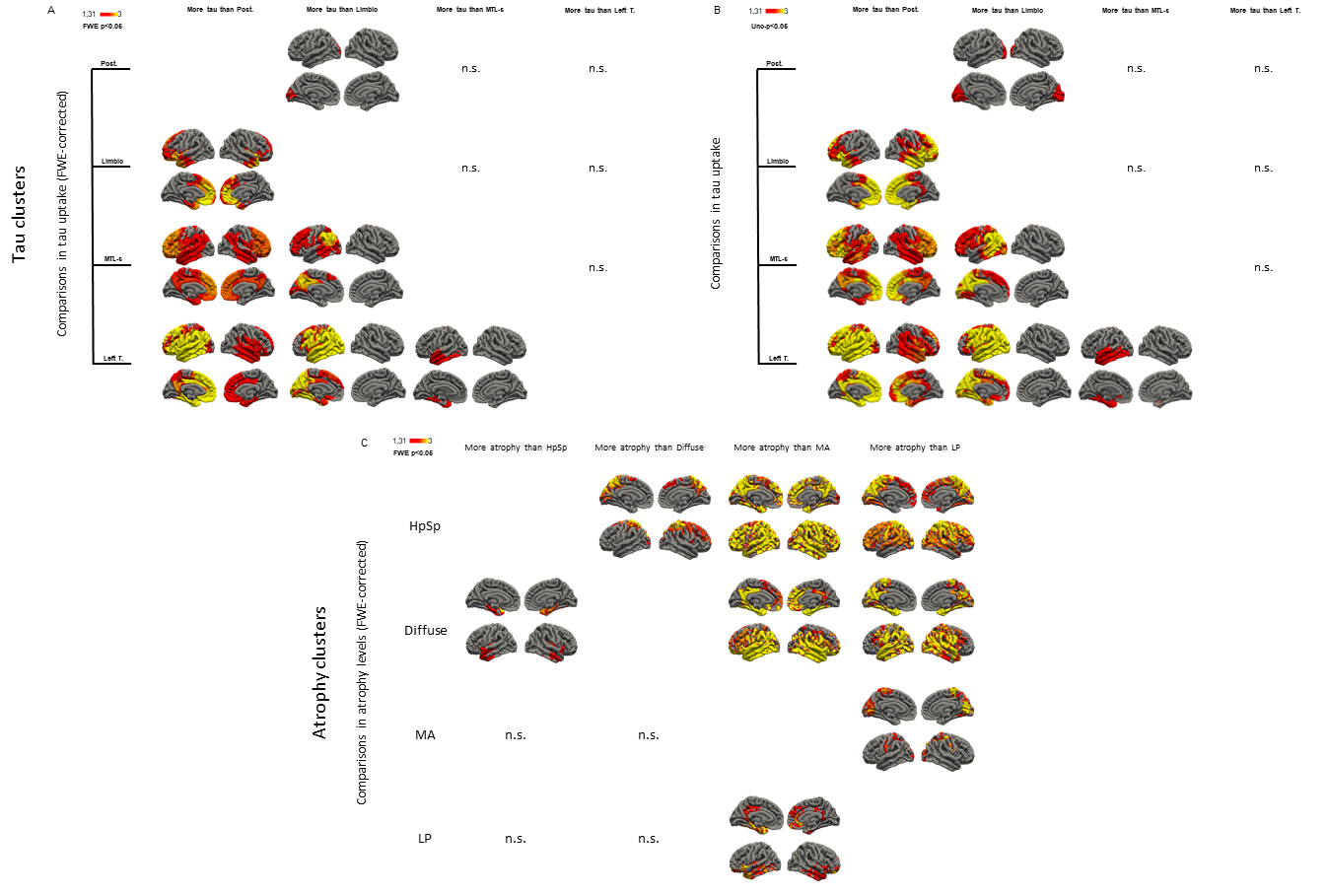


**eFigure 2.** Scatterplots of the correlations between covariance levels of (inverse) cortical volumes and tau PET SUVR among atrophy and tau clusters in their respective modality (i.e., tau PET covariance for tau clusters and inverse covariance in cortical volumes for atrophy clusters). Lines and spearman’s rho are shown in respective scatterplots, when α<0.05 was achieved when adjusted for multiple comparisons using Bonferroni correction. *Bonferroni-p<0.05, ** Bonferroni-p<0.01, ***Bonferroni-p<0.001.

Abbreviations: ADS, Alzheimer’s disease spectrum; MTL-sparing, medial temporal lobe sparing; HpSp, hippocampal-sparing; SUVr, standardized uptake value ratio.

**
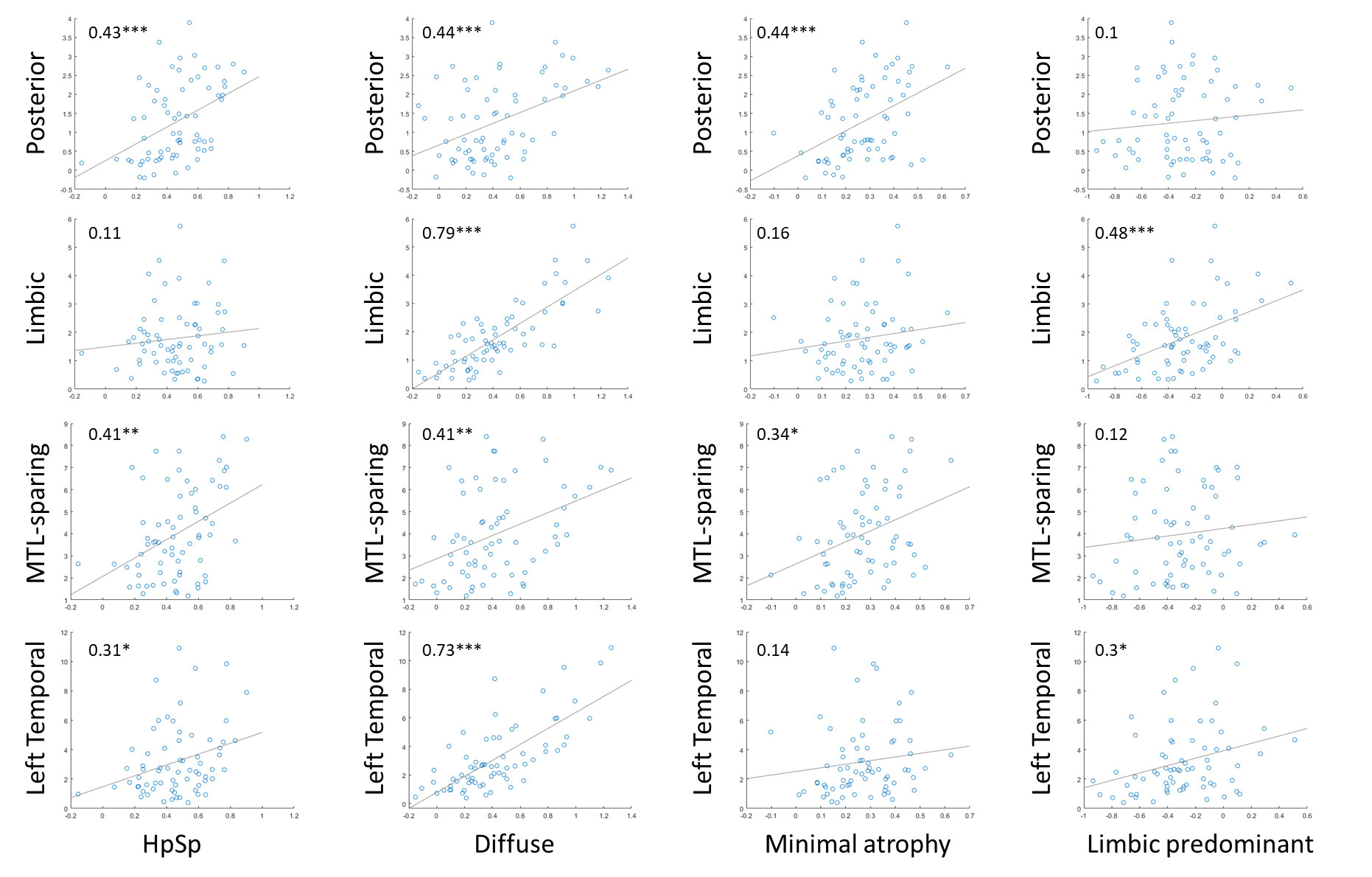
**

**eFigure 3.** Bar charts showing allocations across clusters of tau PET and cerebral atrophy. Numbers and percentages of respective clusters are shown.

**
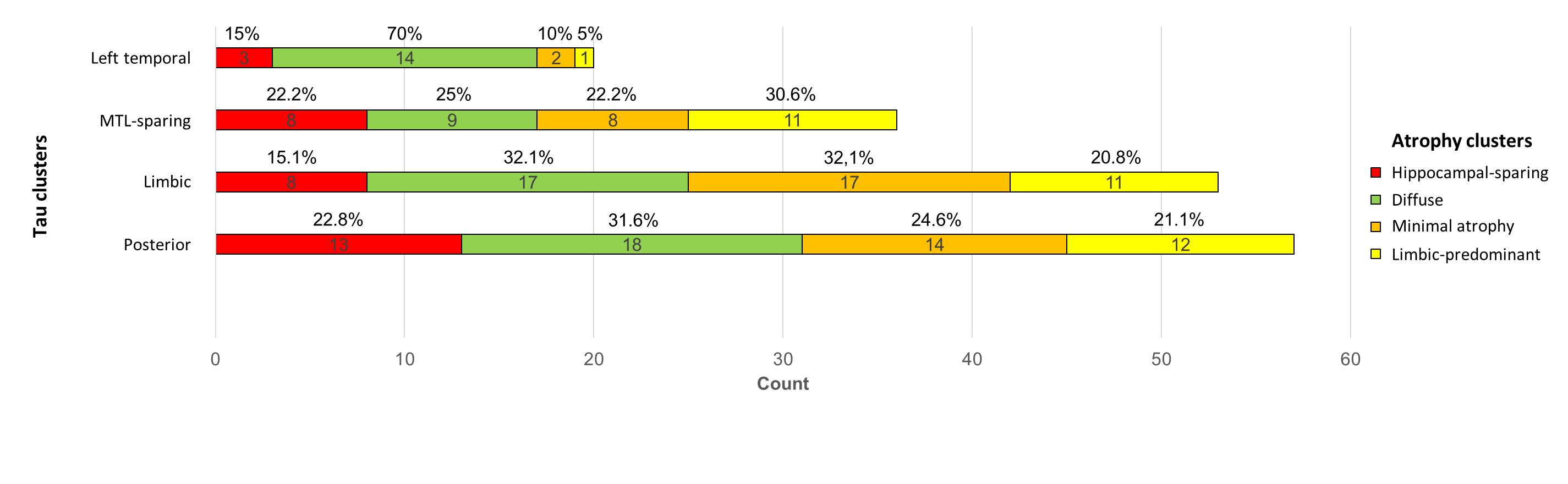
**

**eFigure 4.** Scatter plot of the number of data included in the longitudinal imaging analyses for tau and atrophy clusters, showing the distribution of available data at baseline and follow-up visits among the study groups. Of note, the numbers of available cases can differ from all available baseline data, as only cases with available follow-up data are presented in this figure. Intervals are shown in intervals between the follow-up imaging and baseline Tau PET and MRI for tau and atrophy clusters, respectively.

Abbreviations: ADS, Alzheimer’s disease spectrum; MTL-sparing, medial temporal lobe sparing; Left Temp., left temporal; HpSp, hippocampal-sparing; MA, minimal atrophy; LP, limbic-predominant.


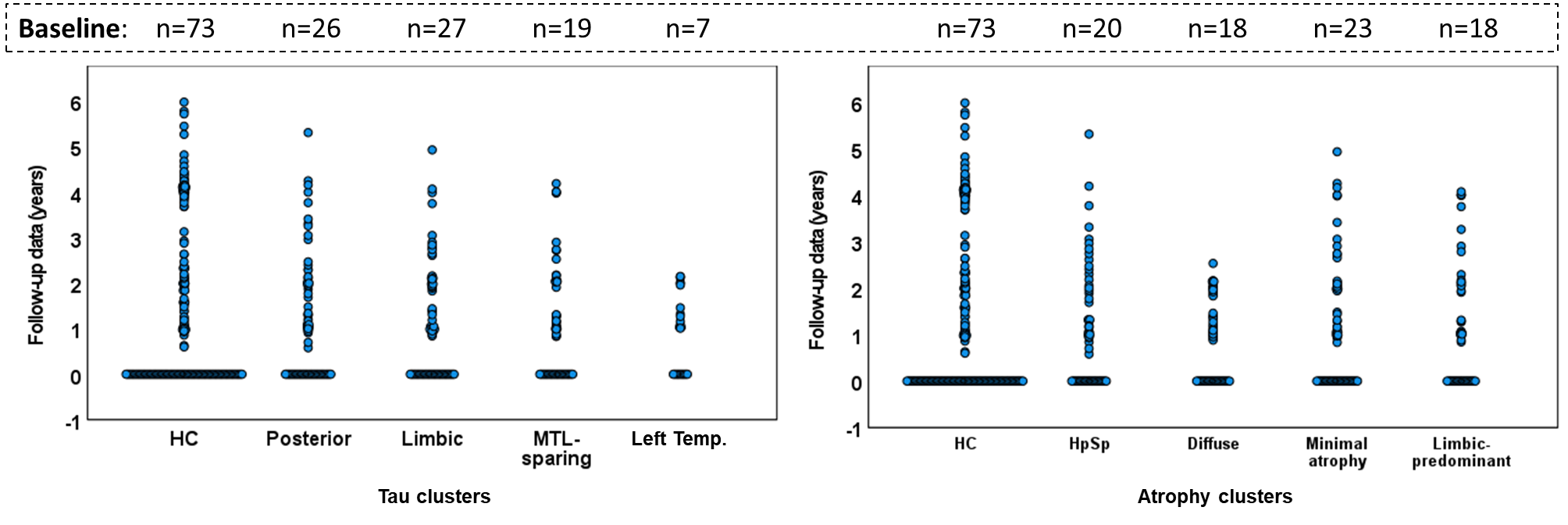


**eFigure 5.** Annual change rates (in z-scores) of tau PET and cortical volumes are shown as group means for tau PET clusters **(A and C)** and atrophy clusters **(B and D)**. The Desikan-Killiany atlas regions are censored when no significant difference among groups were found (significant when uncorrected overall-p<0.05 for a more comprehensive visualisation).

Abbreviations: Left Temp, left temporal; MTL-sparing, medial temporal lobe sparing; HpSp, hippocampal sparing; Limbic-pred., limbic-predominant.


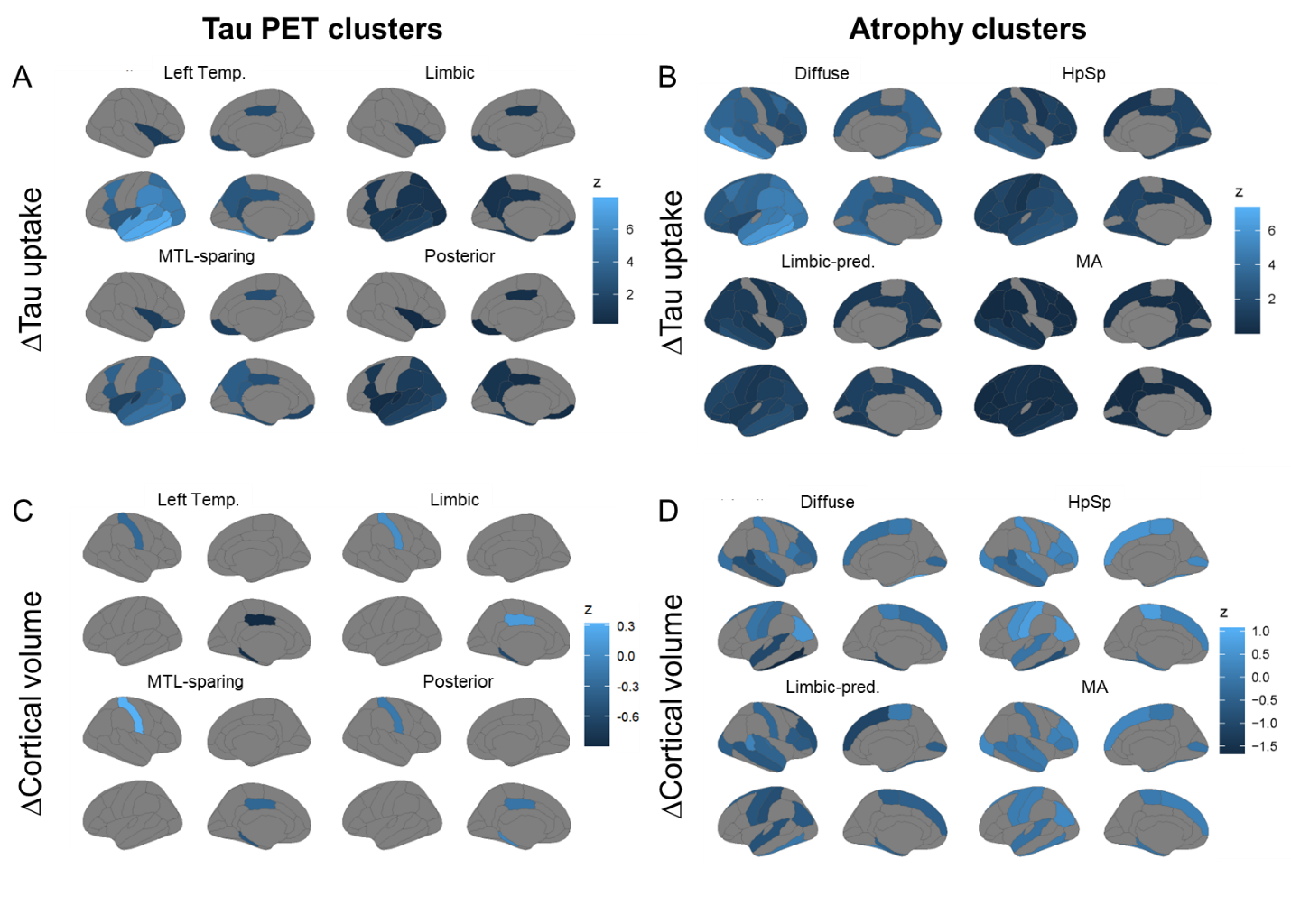


**eFigure 6.** Plots of Rand Indices across different leave-one-out sample sizes for tau (A) and atrophy (B) clusters.


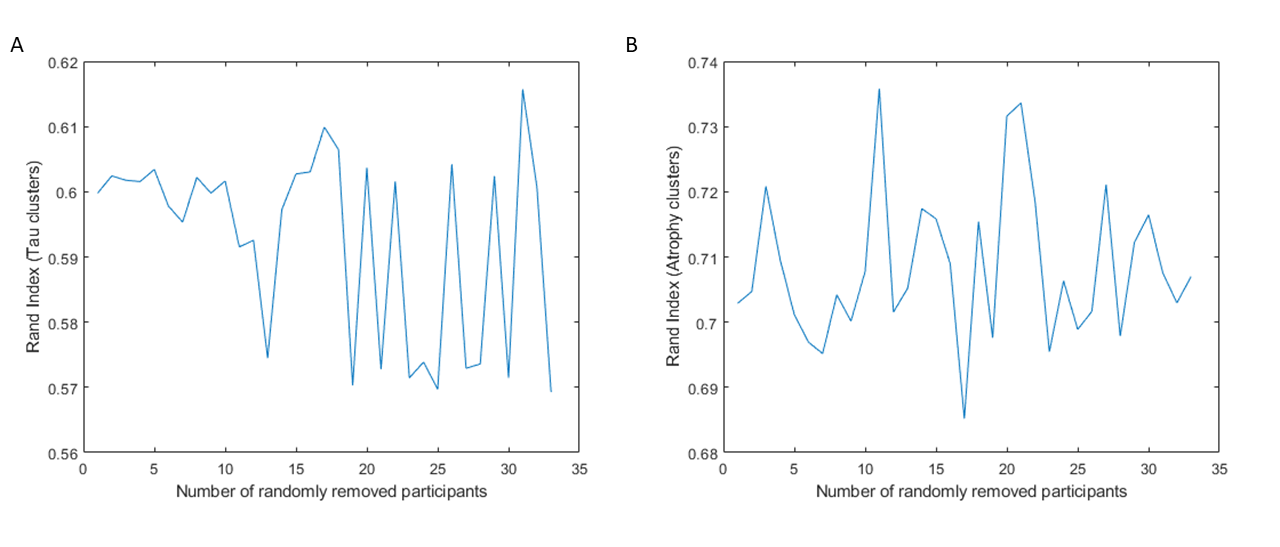


**eTable 1.** Imaging biomarker characteristics of groups.

^a^, versus HC; ^b^, versus Posterior/hippocampal-sparing; ^c^, versus Limbic/Diffuse; ^d^, versus MTL-sparing/minimal atrophy; ^e^, versus left temporal/limbic-predominant. *Bonferroni-p<0.001. Abbreviations: HC, healthy controls; ADS, Alzheimer’s disease spectrum; MTL, medial temporal lobe; SD, standard deviation; ROI, region of interest; Aβ-PET, amyloid-ß PET.

|  |  |  |  |  |  | Tau clusters | | | | | | | | | Atrophy clusters | | | | | | | | | | |
| --- | --- | --- | --- | --- | --- | --- | --- | --- | --- | --- | --- | --- | --- | --- | --- | --- | --- | --- | --- | --- | --- | --- | --- | --- | --- |
|  | HC (N=154) | | ADS (N=166) | |  | Posterior (N=57) | | Limbic (N=53) | | MTL-sparing (N=36) | | Left Temporal (N=20) | |  | Hippocampal-sparing (N=32) | | Diffuse (N=58) | | Minimal atrophy (N=41) | | | Limbic-predominant (N=35) | | |  |
|  | **No.** | **Mean** | **No.** | **Mean** | **p** | **No.** | **Mean** | **No.** | **Mean** | **No** | **Mean** | **No.** | **Mean** | **p(overall)** | **No.** | **Mean** | **No.** | **Mean** | **No.** | **Mean** | **No.** | | **Mean** | **p(overall)** | |
| Tau-PET uptake meta ROI, SUVr | 154 | 1.17 ± 0.07 | 166 | 1.51 ± 0.36 | <0.001 | 57 | 1.38 ^a*,e^ ± 0.27 | 53 | 1.51 ^a*^ ± 0.33 | 36 | 1.59 ^a*^ ± 0.38 | 20 | 1.7 ^a*,b^ ± 0.48 | <0.001 | 32 | 1.46 ^a*,c^ ± 0.33 | 58 | 1.67 ^a*,b,d*,e*^ ± 0.4 | 41 | 1.39 ^a,c*^ ± 0.27 | 35 | | 1.42 ^c*^ ± 0.31 | <0.001 | |
| Cortical/Hippocampus volume ratio | 154 | 58.84 ± 5.99 | 166 | 64.15 ± 8.94 | <0.001 | 57 | 63.2 ^a*^ ± 8.71 | 53 | 66.94 ^a*^ ± 8.82 | 36 | 63.6 ^a*^ ± 8.1 | 20 | 64 ^a*^ ± 8.39 | <0.001 | 32 | 62.61 ^c^ ± 8.02 | 38 | 67.29 ^a*,b,d^ ± 9.7 | 41 | 61.11 ^c,e^ ± 7.35 | 35 | | 65.97 ^a*,d^ ± 7.15 | <0.001 | |
| Mean Hippocampus Volumes | 154 | 3763 ± 423 | 166 | 3336 ± 577 | <0.001 | 57 | 3429 ^a*^ ± 523 | 53 | 3253 ^a*^ ± 632 | 36 | 3340 ^a*^ ± 545 | 20 | 3296 ^a*^ ± 605 | <0.001 | 32 | 3303 ^a*^ ± 541 | 58 | 3115 ^a*,d,e^ ± 510 | 41 | 3507 ^c^ ± 623 | 35 | | 3536 ^c^ ± 526 | <0.001 | |
| Aß-PET uptake whole brain, centiloid | 141 | 6.2 ± 10.3 | 123 | 71.4 ± 40.1 | <0.001 | 43 | 61.7 ^a*^ ± 41 | 37 | 78.3 ^a*^ ± 42.5 | 28 | 73.3 ^a*^ ± 40.5 | 15 | 78.4 ^a*^ ± 26.5 | <0.001 | 27 | 63.8 ^a*^ ± 39.5 | 44 | 81.3 ^a*,d^ ± 38.7 | 26 | 58.3 ^a*,c^ ± 42.4 | 26 | | 75.4 ^a*^ ± 37.8 | <0.001 | |
| Aß-PET uptake frontal, centiloid | 141 | 3.1 ± 11 | 123 | 72.5 ± 41.7 | <0.001 | 43 | 60.9 ^a*,c^ ± 43.4 | 37 | 81.5 ^a*,b^ ± 43.6 | 28 | 77 ^a*^ ± 40.6 | 15 | 75 ^a*^ ± 27.5 | <0.001 | 27 | 62.6 ^a*^ ± 39.9 | 44 | 82.18 ^a*,d^ ± 39.55 | 26 | 61.2 ^a*,c^ ± 45.2 | 26 | | 77.4 ^a*^ ± 40.9 | <0.001 | |
| Aß-PET uptake cingulate, centiloid | 141 | 19.8 ± 13 | 123 | 86 ± 42.3 | <0.001 | 43 | 75.9 ^a*,c^ ± 41.7 | 37 | 95.4 ^a*,b^ ± 44.6 | 28 | 90.4 ^a*^ ± 44.6 | 15 | 86.7 ^a*^ ± 26 | <0.001 | 27 | 80.3 ^a*^ ± 44.5 | 44 | 92.16 ^a*^ ± 39.7 | 26 | 75.4 ^a*^ ± 46.5 | 26 | | 91.9 ^a*^ ± 39.4 | <0.001 | |
| Aß-PET uptake parietal, centiloid | 141 | 5.7 ± 11.8 | 123 | 72.5 ± 40.8 | <0.001 | 43 | 65 ^a*,c^ ± 41.3 | 37 | 76.8 ^a*^ ± 44.1 | 28 | 72 ^a*^ ± 40.6 | 15 | 84.4 ^a*^ ± 28.6 | <0.001 | 27 | 65.8 ^a*^ ± 39.4 | 44 | 85.8 ^a*,d^ ± 40.7 | 26 | 56.9 ^a*,c^ ± 41.4 | 26 | | 72.4 ^a*^ ± 36.6 | <0.001 | |
| Aß-PET uptake temporal, centiloid | 141 | -3.7 ± 10.4 | 123 | 54.5 ± 40.4 | <0.001 | 43 | 46.1 ^a*^ ± 41 | 37 | 59.7 ^a*^ ± 42 | 28 | 53.6 ^a*^ ± 40.1 | 15 | 67.6 ^a*^ ± 32.4 | <0.001 | 27 | 46.6 ^a*^ ± 38.62 | 44 | 65.1 ^a*,d^ ± 40.4 | 26 | 3.8 ^a*,c^ ± 40.2 | 26 | | 59.7 ^a*^ ± 38.3 | <0.001 | |
|  |  |  |  |  |  |  |  |  |  |  |  |  |  |  |  |  |  |  |  |  |  | |  |  | |

**eTable 2.** Annual change rates (z-scores) of cortical tau PET uptake (SUVr) in tau/atrophy clusters. Cortical regions defined in Desikan-Killiany Atlas are presented. Significant results are indicated in bold, when p-FDR<0.05.

^a^, versus Posterior/hippocampal-sparing; ^b^, versus Limbic/Diffuse; ^c^, versus MTL-sparing/minimal atrophy; ^d^, versus left temporal/limbic-predominant. Abbreviations: SUVr, standardized uptake value ratio; FDR, false discovery rate; MTL, medial temporal lobe; HpSp, Hippocampal-sparing; SD, standard deviation; LH, left hemisphere; RH, right hemisphere; STS, Superior Temporal Sulcus.

|  | **Tau clusters** | | | | | | | |  |  | **Atrophy clusters** | | | | | | | |  |  |
| --- | --- | --- | --- | --- | --- | --- | --- | --- | --- | --- | --- | --- | --- | --- | --- | --- | --- | --- | --- | --- |
|  | **Posterior** | | **Limbic** | | **MTL-sparing** | | **Left temporal** | |  |  | **HpSp** | | **Diffuse** | | **Minimal atrophy** | | **Limbic pred.** | |  |  |
|  | Mean | SD | Mean | SD | Mean | SD | Mean | SD | p-overall | p-FDR | Mean | SD | Mean | SD | Mean | SD | Mean | SD | p-overall | p-FDR |
| lh_bankssts_suvr | **1.02** | **3.20** | **0.81** | **1.71** | **3.69** | **4.51** | **5.63** | **4.67** | **0.012** | **0.04** | **2.34** | **3.19** | **4.64^c,d^** | **4.25** | **0.06^b^** | **1.38** | **1.44^b^** | **4.02** | **0.0001** | **0.001** |
| lh_caudalanteriorcingulate_suvr | 0.04 | 1.14 | 0.42 | 0.92 | 0.81 | 1.74 | 1.57 | 1.51 | 0.082 | 0.12 | 0.39 | 1.25 | 1.01 | 1.09 | 0.22 | 1.13 | 0.44 | 1.77 | 0.0994 | 0.109 |
| lh_caudalmiddlefrontal_suvr | 0.86 | 2.48 | 0.89 | 1.19 | 3.51 | 4.77 | 4.26 | 4.03 | 0.045 | 0.08 | **1.66** | **2.21** | **4.13^c,d^** | **3.77** | **0.24^b^** | **1.43** | **1.67^b^** | **4.24** | **0.0006** | **0.002** |
| lh_cuneus_suvr | 1.79 | 4.16 | 0.32 | 0.91 | 2.38 | 2.75 | 2.52 | 3.05 | 0.088 | 0.12 | **1.83** | **2.39** | **3.37^c,d^** | **4.77** | **0.21^b^** | **0.99** | **0.88^b^** | **2.17** | **0.0033** | **0.005** |
| lh_entorhinal_suvr | 1.04 | 1.82 | 1.49 | 1.30 | 2.21 | 1.55 | 2.55 | 2.21 | 0.041 | 0.08 | **1.51** | **1.78** | **2.96^c,d^** | **1.27** | **0.90^b^** | **1.38** | **1.28^b^** | **1.61** | **0.0006** | **0.002** |
| lh_frontalpole_suvr | 0.58 | 1.23 | 0.78 | 1.49 | 1.94 | 3.55 | 2.19 | 3.38 | 0.432 | 0.43 | **0.95** | **1.67** | **2.05^c^** | **2.15** | **0.40^b^** | **1.26** | **1.28** | **3.66** | **0.0161** | **0.020** |
| lh_fusiform_suvr | 1.80 | 4.01 | 1.01 | 1.71 | 3.46 | 4.05 | 7.40 | 5.30 | 0.02 | 0.05 | **2.15** | **3.42** | **5.95^c,d^** | **4.61** | **0.77^b^** | **1.78** | **1.32^b^** | **3.73** | **0.0001** | **0.001** |
| lh_inferiorparietal_suvr | **1.32** | **3.09** | **0.61^c,d^** | **1.25** | **4.09^b^** | **4.54** | **5.05^b^** | **4.26** | **0.005** | **0.04** | **2.14** | **2.91** | **4.76^c,d^** | **4.20** | **0.30^b^** | **1.31** | **1.58^b^** | **3.95** | **0.0002** | **0.001** |
| lh_inferiortemporal_suvr | **1.90^d^** | **3.98** | **1.58** | **2.30** | **4.46** | **5.07** | **7.94^a^** | **5.36** | **0.01** | **0.04** | **2.69** | **3.78** | **6.58^c,d^** | **4.86** | **0.96^b^** | **1.99** | **2.10^b^** | **4.61** | **0.0003** | **0.001** |
| lh_insula_suvr | **0.22^d^** | **1.63** | **0.92** | **1.02** | **1.56** | **2.16** | **2.87^a^** | **3.04** | **0.014** | **0.04** | **1.22** | **2.22** | **1.80^d^** | **1.68** | **0.36** | **1.18** | **0.85^b^** | **2.23** | **0.0284** | **0.035** |
| lh_isthmuscingulate_suvr | **0.60^c^** | **2.25** | **0.73** | **1.21** | **3.03^a^** | **3.20** | **2.19** | **2.44** | **0.009** | **0.04** | **1.71** | **2.00** | **2.76^c,d^** | **2.39** | **0.06^b^** | **1.37** | **1.27^b^** | **3.23** | **0.0006** | **0.002** |
| lh_lateraloccipital_suvr | **2.24** | **4.21** | **0.35^c,d^** | **1.09** | **3.03^b^** | **3.73** | **4.86^b^** | **5.22** | **0.006** | **0.04** | **2.35** | **3.80** | **4.41^c,d^** | **4.63** | **0.48^b^** | **1.29** | **1.20^b^** | **3.37** | **0.0009** | **0.002** |
| lh_lateralorbitofrontal_suvr | 0.23 | 1.48 | 1.06 | 1.38 | 2.19 | 3.71 | 2.37 | 3.39 | 0.068 | 0.10 | **1.33** | **1.96** | **2.15^c,d^** | **2.18** | **0.24^b^** | **1.45** | **1.22^b^** | **3.72** | **0.0054** | **0.007** |
| lh_lingual_suvr | 1.49 | 3.52 | 0.41 | 1.11 | 2.28 | 3.20 | 3.50 | 3.72 | 0.132 | 0.16 | **1.52** | **2.18** | **3.59^c,d^** | **4.20** | **0.24^b^** | **1.14** | **0.96^b^** | **2.90** | **0.0029** | **0.005** |
| lh_medialorbitofrontal_suvr | 0.16 | 1.55 | 1.04 | 1.32 | 1.74 | 2.94 | 2.72 | 3.56 | 0.027 | 0.07 | 1.18 | 1.76 | 1.80 | 2.31 | 0.45 | 1.58 | 1.01 | 3.09 | 0.0986 | 0.109 |
| lh_middletemporal_suvr | **1.52^d^** | **3.70** | **1.40** | **2.07** | **4.35** | **5.15** | **7.49^a^** | **5.78** | **0.008** | **0.04** | **2.53** | **3.66** | **6.01^c,d^** | **4.85** | **0.61^b^** | **1.78** | **2.21^b^** | **4.84** | **0.0002** | **0.001** |
| lh_paracentral_suvr | 0.23 | 1.11 | 0.16 | 1.07 | 1.30 | 2.42 | 1.63 | 2.09 | 0.222 | 0.23 | 0.16 | 0.91 | 1.47 | 1.82 | 0.44 | 1.77 | 0.36 | 1.84 | 0.0542 | 0.064 |
| lh_parahippocampal_suvr | 1.12 | 2.32 | 1.37 | 1.53 | 2.21 | 2.32 | 3.41 | 2.39 | 0.043 | 0.08 | **1.43** | **1.87** | **3.41^c,d^** | **2.02** | **0.94^b^** | **1.60** | **1.16^b^** | **2.41** | **0.0013** | **0.003** |
| lh_parsopercularis_suvr | 0.45 | 2.12 | 0.66 | 1.12 | 2.75 | 4.10 | 3.49 | 3.45 | 0.036 | 0.08 | **1.50** | **2.35** | **2.82^c,d^** | **2.66** | **0.06^b^** | **1.43** | **1.34^b^** | **4.01** | **0.0012** | **0.003** |
| lh_parsorbitalis_suvr | 0.26 | 1.35 | 0.65 | 1.26 | 2.22 | 4.28 | 2.09 | 2.84 | 0.178 | 0.19 | **1.06** | **2.33** | **2.08^c,d^** | **1.95** | **0.07^b^** | **1.26** | **1.17^b^** | **4.02** | **0.0017** | **0.003** |
| lh_parstriangularis_suvr | 0.37 | 1.91 | 0.75 | 1.27 | 2.64 | 4.19 | 2.47 | 3.15 | 0.057 | 0.09 | **1.23** | **2.33** | **2.54^c,d^** | **2.47** | **0.16^b^** | **1.53** | **1.29^b^** | **3.97** | **0.0029** | **0.005** |
| lh_pericalcarine_suvr | 1.18 | 2.26 | 0.31 | 0.82 | 1.33 | 1.63 | 1.29 | 2.09 | 0.135 | 0.16 | 1.15 | 1.31 | 1.67 | 2.79 | 0.30 | 0.86 | 0.76 | 1.40 | 0.1808 | 0.186 |
| lh_postcentral_suvr | 0.79 | 2.02 | 0.31 | 0.98 | 1.82 | 3.24 | 3.91 | 4.12 | 0.121 | 0.15 | **0.60** | **1.44** | **3.08^c,d^** | **3.37** | **0.43^b^** | **1.50** | **0.75^b^** | **2.79** | **0.0088** | **0.011** |
| lh_posteriorcingulate_suvr | **0.32^c^** | **1.74** | **0.70** | **1.37** | **2.43^a^** | **3.23** | **2.79** | **2.57** | **0.008** | **0.04** | **1.21** | **1.83** | **2.53^c,d^** | **2.06** | **0.12^b^** | **1.43** | **1.13^b^** | **3.30** | **0.0016** | **0.003** |
| lh_precentral_suvr | 0.89 | 2.20 | 0.45 | 1.14 | 2.07 | 3.05 | 4.09 | 4.35 | 0.139 | 0.16 | **1.00** | **1.74** | **3.17^c,d^** | **3.32** | **0.40^b^** | **1.63** | **0.95^b^** | **2.83** | **0.0055** | **0.007** |
| lh_precuneus_suvr | **0.68^c^** | **2.56** | **0.57** | **1.07** | **3.28^a^** | **3.68** | **2.83** | **2.99** | **0.01** | **0.04** | **1.60** | **2.11** | **3.30^c,d^** | **3.16** | **0.003^b^** | **1.23** | **1.31^b^** | **3.49** | **0.0001** | **0.001** |
| lh_rostralanteriorcingulate_suvr | -0.04 | 1.13 | 0.51 | 0.89 | 0.74 | 1.41 | 1.72 | 2.37 | 0.056 | 0.09 | 0.53 | 1.30 | 0.79 | 1.57 | 0.33 | 1.16 | 0.36 | 1.42 | 0.6167 | 0.617 |
| lh_rostralmiddlefrontal_suvr | 0.44 | 1.82 | 0.84 | 1.75 | 2.86 | 4.68 | 1.81 | 2.49 | 0.115 | 0.15 | **1.26** | **2.10** | **2.62^c,d^** | **2.30** | **0.04^b^** | **1.43** | **1.56^b^** | **4.72** | **0.0022** | **0.004** |
| lh_superiorfrontal_suvr | 0.39 | 1.60 | 0.85 | 1.35 | 2.55 | 4.21 | 3.06 | 3.16 | 0.169 | 0.19 | 1.08 | 1.64 | 2.73 | 2.41 | 0.29 | 1.63 | 1.43 | 4.18 | 0.0050 | 0.007 |
| lh_superiorparietal_suvr | 1.35 | 2.95 | 0.58 | 1.09 | 3.17 | 3.95 | 4.07 | 4.17 | 0.043 | 0.08 | **1.70** | **2.17** | **4.23^c,d^** | **4.00** | **0.40^b^** | **1.24** | **1.13^b^** | **3.40** | **0.0001** | **0.001** |
| lh_superiortemporal_suvr | **0.81^d^** | **2.64** | **0.92** | **1.52** | **3.17** | **4.39** | **6.66^a^** | **5.90** | **0.004** | **0.04** | **1.89** | **2.97** | **4.54^c,d^** | **4.39** | **0.33^b^** | **1.42** | **1.42^b^** | **4.27** | **0.0004** | **0.001** |
| lh_supramarginal_suvr | **1.12^d^** | **3.34** | **0.74** | **1.28** | **3.27** | **4.12** | **5.60^a^** | **4.74** | **0.012** | **0.04** | **1.81** | **2.44** | **4.76^c,d^** | **4.49** | **0.23^b^** | **1.31** | **1.29^b^** | **3.71** | **0.0001** | **0.001** |
| lh_temporalpole_suvr | **0.85^d^** | **2.09** | **1.67** | **1.91** | **1.80** | **1.78** | **4.57^a^** | **3.44** | **0.007** | **0.04** | **1.46^b^** | **2.12** | **3.64^a,c,d^** | **2.58** | **0.71^b^** | **1.17** | **1.24^b^** | **2.23** | **0.0005** | **0.002** |
| lh_transversetemporal_suvr | 0.17 | 1.62 | 0.21 | 1.20 | 1.16 | 1.97 | 2.74 | 3.63 | 0.042 | 0.08 | 0.80 | 1.87 | 1.56 | 2.51 | -0.09 | 1.03 | 0.50 | 2.07 | 0.1194 | 0.127 |
| rh_bankssts_suvr | 0.68 | 2.53 | 1.21 | 2.67 | 3.14 | 4.12 | 2.08 | 2.87 | 0.137 | 0.32 | **2.44** | **3.43** | **3.08^c,d^** | **2.91** | **0.03^b^** | **1.52** | **1.09^b^** | **3.76** | **0.0006** | **0.003** |
| rh_caudalanteriorcingulate_suvr | -0.07 | 1.29 | 0.60 | 1.20 | 0.65 | 1.78 | 1.30 | 1.16 | 0.051 | 0.32 | 0.24 | 1.38 | 0.99 | 1.10 | 0.34 | 1.47 | 0.29 | 1.66 | 0.1506 | 0.171 |
| rh_caudalmiddlefrontal_suvr | 0.66 | 1.94 | 1.08 | 1.91 | 3.18 | 4.80 | 1.86 | 3.33 | 0.392 | 0.47 | **1.44** | **1.95** | **3.53^c,d^** | **3.86** | **0.25^b^** | **1.73** | **1.22^b^** | **3.79** | **0.0013** | **0.004** |
| rh_cuneus_suvr | 2.45 | 5.01 | 0.46 | 1.02 | 1.91 | 2.74 | 1.07 | 1.57 | 0.269 | 0.40 | **1.63** | **2.63** | **3.41^c,d^** | **5.22** | **0.47^b^** | **1.22** | **0.84^b^** | **2.83** | **0.0031** | **0.007** |
| rh_entorhinal_suvr | 1.10 | 1.83 | 1.53 | 1.59 | 1.83 | 1.42 | 1.88 | 2.06 | 0.331 | 0.43 | **1.35** | **1.90** | **2.57^c^** | **1.19** | **0.98^b^** | **1.59** | **1.21** | **1.57** | **0.0096** | **0.017** |
| rh_frontalpole_suvr | 0.28 | 1.37 | 0.91 | 1.63 | 1.73 | 3.68 | 1.23 | 2.43 | 0.447 | 0.49 | **1.02** | **1.92** | **1.74** | **1.87** | **0.31** | **1.45** | **0.80** | **3.61** | **0.0377** | **0.049** |
| rh_fusiform_suvr | 3.10 | 5.84 | 2.00 | 2.62 | 3.35 | 4.16 | 5.92 | 5.34 | 0.245 | 0.38 | **2.85** | **4.03** | **6.77^c,d^** | **5.69** | **1.43^b^** | **2.79** | **1.56^b^** | **3.63** | **0.0004** | **0.003** |
| rh_inferiorparietal_suvr | 1.03 | 2.80 | 0.83 | 1.87 | 2.98 | 3.70 | 1.44 | 1.99 | 0.142 | 0.32 | **1.77** | **2.20** | **3.42^c,d^** | **3.24** | **0.05^b^** | **1.34** | **0.99^b^** | **3.30** | **0.0002** | **0.003** |
| rh_inferiortemporal_suvr | 2.74 | 5.05 | 2.94 | 3.74 | 4.50 | 5.46 | 6.39 | 5.79 | 0.141 | 0.32 | **3.36** | **4.39** | **7.36^c,d^** | **5.84** | **1.78^b^** | **2.95** | **2.24^b^** | **4.46** | **0.0011** | **0.004** |
| rh_insula_suvr | 0.12 | 1.54 | 1.19 | 1.43 | 1.25 | 1.59 | 1.20 | 1.64 | 0.014 | 0.32 | 1.14 | 1.81 | 1.29 | 1.32 | 0.53 | 1.46 | 0.52 | 1.67 | 0.1598 | 0.175 |
| rh_isthmuscingulate_suvr | 0.69 | 2.23 | 1.06 | 1.72 | 2.96 | 3.47 | 1.72 | 2.28 | 0.069 | 0.32 | **1.62** | **1.77** | **2.95^c,d^** | **2.45** | **0.32^b^** | **1.78** | **1.22^b^** | **3.52** | **0.0011** | **0.004** |
| rh_lateraloccipital_suvr | 3.11 | 5.70 | 0.40 | 1.25 | 2.33 | 3.58 | 2.01 | 2.59 | 0.172 | 0.33 | **2.07** | **3.33** | **4.31^c,d^** | **5.67** | **0.61^b^** | **2.16** | **0.94^b^** | **3.48** | **0.0011** | **0.004** |
| rh_lateralorbitofrontal_suvr | 0.14 | 1.49 | 1.35 | 1.71 | 1.89 | 3.05 | 1.29 | 2.43 | 0.04 | 0.32 | 1.34 | 1.93 | 1.97 | 2.10 | 0.36 | 1.65 | 0.81 | 2.83 | 0.0138 | 0.022 |
| rh_lingual_suvr | 2.37 | 5.03 | 0.58 | 1.27 | 1.81 | 3.30 | 1.59 | 2.55 | 0.721 | 0.72 | **1.34** | **2.39** | **3.65^c,d^** | **5.26** | **0.53^b^** | **1.53** | **0.99^b^** | **3.54** | **0.0019** | **0.005** |
| rh_medialorbitofrontal_suvr | 0.02 | 1.61 | 1.13 | 1.56 | 1.41 | 2.61 | 1.86 | 3.68 | 0.027 | 0.32 | 0.98 | 1.87 | 1.66 | 2.35 | 0.44 | 1.72 | 0.63 | 2.68 | 0.0814 | 0.099 |
| rh_middletemporal_suvr | 1.37 | 3.18 | 2.02 | 3.06 | 3.41 | 4.48 | 4.15 | 4.43 | 0.114 | 0.32 | **2.57** | **3.47** | **4.91^c,d^** | **4.03** | **0.74^b^** | **2.13** | **1.51^b^** | **3.85** | **0.0003** | **0.003** |
| rh_paracentral_suvr | 0.19 | 1.28 | 0.18 | 1.14 | 1.00 | 1.93 | 1.23 | 1.93 | 0.401 | 0.47 | 0.05 | 1.12 | 1.27 | 1.66 | 0.35 | 1.61 | 0.30 | 1.44 | 0.1001 | 0.117 |
| rh_parahippocampal_suvr | 1.08 | 2.39 | 1.43 | 1.89 | 1.79 | 2.03 | 2.97 | 2.71 | 0.205 | 0.35 | **1.41^b^** | **2.19** | **3.35^a,c,d^** | **1.82** | **0.81^b^** | **1.87** | **0.79^b^** | **2.03** | **0.0003** | **0.003** |
| rh_parsopercularis_suvr | 0.20 | 1.52 | 0.88 | 1.39 | 2.57 | 4.15 | 0.91 | 1.42 | 0.174 | 0.33 | **1.36** | **2.07** | **1.88^c,d^** | **2.00** | **0.09^b^** | **1.42** | **1.17^b^** | **3.94** | **0.0042** | **0.008** |
| rh_parsorbitalis_suvr | 0.13 | 1.15 | 0.80 | 1.40 | 2.02 | 3.87 | 0.74 | 1.91 | 0.292 | 0.41 | **0.96** | **2.36** | **1.78^c,d^** | **2.06** | **0.10^b^** | **1.22** | **0.83^b^** | **3.21** | **0.0162** | **0.025** |
| rh_parstriangularis_suvr | 0.25 | 1.34 | 0.86 | 1.45 | 2.48 | 3.87 | 0.63 | 1.31 | 0.123 | 0.32 | **1.05** | **2.17** | **1.87^c^** | **1.83** | **0.21** | **1.40** | **1.21^b^** | **3.60** | **0.0188** | **0.028** |
| rh_pericalcarine_suvr | 1.88 | 3.38 | 0.32 | 1.14 | 1.16 | 2.06 | 0.40 | 1.16 | 0.4 | 0.47 | 1.17 | 2.13 | 1.91 | 3.41 | 0.50 | 1.44 | 0.72 | 2.25 | 0.1685 | 0.179 |
| rh_postcentral_suvr | 0.52 | 1.31 | 0.52 | 1.17 | 1.32 | 2.22 | 1.15 | 1.42 | 0.519 | 0.55 | 0.47 | 1.19 | 1.74 | 1.91 | 0.41 | 1.34 | 0.58 | 1.51 | 0.0629 | 0.079 |
| rh_posteriorcingulate_suvr | 0.37 | 1.79 | 0.86 | 1.57 | 2.29 | 3.39 | 2.10 | 2.16 | 0.049 | 0.32 | **1.13** | **1.79** | **2.48^c,d^** | **2.01** | **0.24^b^** | **1.66** | **1.01^b^** | **3.31** | **0.0025** | **0.006** |
| rh_precentral_suvr | 0.69 | 1.74 | 0.53 | 1.25 | 1.68 | 2.68 | 1.53 | 2.75 | 0.579 | 0.60 | **0.76** | **1.58** | **2.27** | **2.61** | **0.35** | **1.43** | **0.60** | **1.85** | **0.0356** | **0.048** |
| rh_precuneus_suvr | 0.93 | 2.90 | 0.85 | 1.58 | 3.00 | 3.82 | 1.60 | 2.31 | 0.139 | 0.32 | **1.56** | **1.99** | **3.36^c,d^** | **3.35** | **0.18^b^** | **1.51** | **1.09^b^** | **3.53** | **0.0006** | **0.003** |
| rh_rostralanteriorcingulate_suvr | -0.03 | 1.01 | 0.51 | 0.81 | 0.36 | 1.05 | 1.21 | 2.20 | 0.083 | 0.32 | 0.19 | 1.08 | 0.59 | 1.46 | 0.38 | 0.98 | 0.29 | 1.10 | 0.7097 | 0.710 |
| rh_rostralmiddlefrontal_suvr | 0.21 | 1.39 | 1.06 | 2.14 | 2.90 | 4.75 | 1.04 | 2.56 | 0.226 | 0.37 | **1.22** | **2.19** | **2.48^c^** | **3.00** | **0.11^b^** | **1.54** | **1.38** | **4.46** | **0.0207** | **0.029** |
| rh_superiorfrontal_suvr | 0.21 | 1.45 | 1.02 | 1.85 | 2.13 | 3.65 | 1.68 | 2.63 | 0.305 | 0.41 | **0.76** | **1.48** | **2.53^c,d^** | **2.78** | **0.38^b^** | **1.86** | **0.88^b^** | **3.12** | **0.0098** | **0.017** |
| rh_superiorparietal_suvr | 1.41 | 3.06 | 0.65 | 1.30 | 2.40 | 3.58 | 1.15 | 1.91 | 0.449 | 0.49 | **1.39** | **1.77** | **3.19^c,d^** | **3.49** | **0.32^b^** | **1.28** | **0.84^b^** | **3.19** | **0.0009** | **0.004** |
| rh_superiortemporal_suvr | 0.54 | 2.13 | 1.34 | 2.20 | 2.12 | 3.26 | 3.08 | 3.86 | 0.122 | 0.32 | **1.82** | **2.95** | **2.89^c,d^** | **2.84** | **0.42^b^** | **1.54** | **0.79^b^** | **2.89** | **0.0035** | **0.007** |
| rh_supramarginal_suvr | 0.78 | 2.56 | 0.99 | 1.73 | 2.59 | 3.61 | 1.62 | 1.88 | 0.207 | 0.35 | **1.53** | **1.89** | **3.11^c,d^** | **3.17** | **0.15^b^** | **1.30** | **0.98^b^** | **3.17** | **0.0006** | **0.003** |
| rh_temporalpole_suvr | 0.60 | 1.84 | 1.67 | 1.84 | 1.25 | 1.18 | 2.57 | 3.19 | 0.082 | 0.32 | **1.25** | **2.19** | **2.72^c,d^** | **1.99** | **0.72^b^** | **1.55** | **0.67^b^** | **1.18** | **0.0018** | **0.005** |
| rh_transversetemporal_suvr | -0.10 | 1.40 | 0.49 | 1.29 | 0.61 | 1.54 | 0.34 | 1.42 | 0.155 | 0.33 | 0.62 | 1.48 | 0.55 | 1.36 | 0.05 | 1.25 | 0.09 | 1.55 | 0.2012 | 0.207 |

**eTable 3.** Annual change rates (z-scores) of cortical volumes in tau/atrophy clusters. Cortical regions defined in Desikan-Killiany Atlas are presented. Significant results are indicated in bold, when p-FDR<0.05.

^a^, versus Posterior/hippocampal-sparing; ^b^, versus Limbic/Diffuse; ^c^, versus MTL-sparing/minimal atrophy; ^d^, versus left temporal/limbic-predominant; when p-Bonferroni<0.05. Abbreviations: FDR, false discovery rate; MTL, medial temporal lobe; HpSp, Hippocampal-sparing; SD, standard deviation; LH, left hemisphere; RH, right hemisphere; STS, Superior Temporal Sulcus.

|  | **Tau clusters** | | | | | | | |  |  | **Atrophy clusters** | | | | | | | |  |  |
| --- | --- | --- | --- | --- | --- | --- | --- | --- | --- | --- | --- | --- | --- | --- | --- | --- | --- | --- | --- | --- |
|  | **Posterior** | | **Limbic** | | **MTL-sparing** | | **Left Temporal** | |  |  | **HpSp** | | **Diffuse** | | **Minimal atrophy** | | **Limbic-pred.** | |  |  |
|  | Mean | SD | Mean | SD | Mean | SD | Mean | SD | p-overall | p-FDR | Mean | SD | Mean | SD | Mean | SD | Mean | SD | p-overall | p-FDR |
| lh_bankssts_vol | 0.01 | 0.93 | -0.25 | 1.09 | 0.34 | 0.86 | 0.52 | 0.78 | 0.14 | 0.60 | 0.10 | 0.78 | 0.27 | 0.95 | 0.19 | 1.01 | -0.42 | 1.10 | 0.20 | 0.42 |
| lh_caudalanteriorcingulate_vol | 0.32 | 0.94 | -0.08 | 0.82 | 0.50 | 0.88 | -0.27 | 1.38 | 0.11 | 0.60 | 0.32 | 0.68 | 0.43 | 0.82 | 0.06 | 1.00 | -0.09 | 1.22 | 0.45 | 0.62 |
| lh_caudalmiddlefrontal_vol | -0.22 | 1.31 | -0.29 | 1.08 | -0.65 | 1.15 | -0.05 | 1.39 | 0.70 | 0.81 | -0.32 | 1.03 | -0.61 | 1.45 | -0.55 | 0.82 | 0.21 | 1.39 | 0.34 | 0.50 |
| lh_cuneus_vol | -0.07 | 1.13 | 0.42 | 1.00 | 0.27 | 1.27 | 0.13 | 0.26 | 0.41 | 0.70 | -0.30 | 1.01 | 0.42 | 1.06 | 0.12 | 1.06 | 0.63 | 1.03 | 0.06 | 0.18 |
| lh_entorhinal_vol | -0.14 | 0.82 | -0.45 | 0.97 | -0.53 | 0.72 | -0.54 | 0.75 | 0.44 | 0.70 | 0.001 | 1.04 | -0.80 | 0.74 | -0.15 | 0.54 | -0.64 | 0.82 | 0.03 | 0.10 |
| lh_frontalpole_vol | 0.07 | 1.57 | -0.06 | 0.83 | 0.06 | 0.97 | -0.02 | 1.31 | 0.97 | 0.97 | 0.17 | 1.28 | -0.05 | 1.04 | 0.06 | 1.46 | -0.15 | 0.75 | 0.99 | 0.99 |
| lh_fusiform_vol | -0.17 | 1.15 | -0.20 | 1.08 | -0.73 | 1.36 | -1.07 | 2.06 | 0.32 | 0.70 | **-0.47** | **1.31** | **-1.25^c,d^** | **1.33** | **-0.06^b^** | **1.07** | **0.12^b^** | **1.13** | **0.008** | **0.04** |
| lh_inferiorparietal_vol | 0.24 | 1.35 | -0.08 | 1.08 | 0.51 | 1.38 | 0.49 | 1.49 | 0.42 | 0.70 | **0.63^d^** | **1.14** | **0.55** | **1.51** | **0.30** | **1.08** | **-0.66^a^** | **1.06** | **0.009** | **0.04** |
| lh_inferiortemporal_vol | -0.38 | 1.08 | -0.29 | 1.27 | -1.03 | 1.74 | -1.35 | 2.21 | 0.12 | 0.60 | **-0.83** | **1.30** | **-1.66^c,d^** | **1.44** | **0.02^b^** | **1.05** | **-0.04^b^** | **1.50** | **0.001** | **0.02** |
| lh_insula_vol | 0.02 | 1.21 | 0.63 | 1.19 | 0.55 | 1.25 | 0.27 | 1.58 | 0.30 | 0.70 | 0.45 | 1.19 | 0.83 | 1.21 | 0.05 | 1.41 | 0.26 | 1.09 | 0.33 | 0.50 |
| lh_isthmuscingulate_vol | -0.03 | 1.06 | -0.05 | 1.18 | -0.25 | 1.28 | 0.20 | 1.64 | 0.90 | 0.93 | -0.20 | 1.37 | 0.01 | 1.17 | -0.09 | 1.28 | 0.03 | 0.97 | 0.97 | 0.99 |
| lh_lateraloccipital_vol | -0.68 | 1.17 | -0.04 | 1.26 | -0.33 | 1.39 | -0.66 | 1.09 | 0.51 | 0.76 | -0.65 | 1.19 | -0.70 | 1.40 | -0.24 | 1.23 | 0.08 | 1.16 | 0.31 | 0.50 |
| lh_lateralorbitofrontal_vol | 0.12 | 0.80 | 0.32 | 1.25 | 0.49 | 1.33 | -0.83 | 1.52 | 0.33 | 0.70 | 0.05 | 1.07 | 0.25 | 1.26 | 0.30 | 1.06 | 0.16 | 1.49 | 0.77 | 0.87 |
| lh_lingual_vol | -0.31 | 1.07 | 0.40 | 0.90 | 0.21 | 0.95 | 0.24 | 0.87 | 0.17 | 0.60 | -0.10 | 0.87 | 0.09 | 1.02 | 0.04 | 1.19 | 0.44 | 0.82 | 0.28 | 0.50 |
| lh_medialorbitofrontal_vol | -0.26 | 1.17 | -0.37 | 1.39 | -0.59 | 1.30 | 0.21 | 1.32 | 0.60 | 0.79 | -0.10 | 1.42 | -0.37 | 1.21 | -0.54 | 1.24 | -0.30 | 1.32 | 0.82 | 0.90 |
| lh_middletemporal_vol | -0.38 | 0.99 | -0.61 | 1.23 | -1.31 | 1.79 | -1.46 | 1.92 | 0.17 | 0.60 | -0.86 | 1.38 | -1.43 | 1.52 | -0.21 | 1.06 | -0.78 | 1.56 | 0.06 | 0.18 |
| lh_paracentral_vol | 0.17 | 0.83 | 0.03 | 1.24 | 0.14 | 1.08 | -0.35 | 1.35 | 0.74 | 0.82 | **0.71^d^** | **1.02** | **0.06** | **0.76** | **0.03** | **1.10** | **-0.56^a^** | **1.06** | **0.004** | **0.04** |
| lh_parahippocampal_vol | -0.05 | 0.62 | -0.60 | 0.99 | -0.48 | 0.93 | -0.86 | 0.78 | 0.02 | 0.48 | **-0.18^b^** | **1.10** | **-0.97^a,c^** | **0.82** | **-0.12^b^** | **0.73** | **-0.49** | **0.57** | **0.009** | **0.04** |
| lh_parsopercularis_vol | -0.21 | 0.76 | -0.03 | 1.36 | -0.70 | 1.06 | 0.03 | 0.74 | 0.26 | 0.69 | -0.25 | 1.27 | -0.36 | 0.78 | -0.29 | 1.01 | -0.07 | 1.27 | 0.91 | 0.96 |
| lh_parsorbitalis_vol | 0.24 | 1.06 | 0.54 | 1.13 | 0.67 | 1.46 | 0.31 | 1.31 | 0.79 | 0.84 | 0.40 | 1.41 | 0.59 | 1.29 | 0.30 | 1.14 | 0.57 | 0.99 | 0.68 | 0.81 |
| lh_parstriangularis_vol | 0.07 | 1.37 | -0.14 | 1.19 | 0.06 | 1.28 | 0.21 | 1.08 | 0.71 | 0.81 | 0.47 | 1.82 | 0.12 | 0.94 | -0.09 | 0.76 | -0.50 | 1.12 | 0.23 | 0.43 |
| lh_pericalcarine_vol | 0.21 | 1.13 | -0.43 | 1.09 | -0.32 | 1.14 | -0.48 | 0.92 | 0.18 | 0.60 | -0.02 | 1.11 | -0.45 | 0.97 | 0.01 | 1.36 | -0.41 | 0.92 | 0.16 | 0.37 |
| lh_postcentral_vol | 0.01 | 0.70 | -0.14 | 1.10 | 0.21 | 1.24 | -0.50 | 1.51 | 0.60 | 0.79 | **0.72^b,d^** | **0.82** | **-0.24^a^** | **0.54** | **0.12^d^** | **1.06** | **-0.88^a,c^** | **1.08** | **0.00004** | **0.001** |
| lh_posteriorcingulate_vol | -0.20 | 0.69 | 0.13 | 0.70 | -0.32 | 0.91 | -0.90 | 1.26 | 0.03 | 0.48 | 0.10 | 0.60 | -0.42 | 0.97 | 0.004 | 0.87 | -0.47 | 0.82 | 0.08 | 0.21 |
| lh_precentral_vol | 0.01 | 0.86 | -0.24 | 1.30 | 0.51 | 1.22 | -0.96 | 1.54 | 0.20 | 0.61 | **0.47^d^** | **0.70** | **-0.07** | **1.16** | **0.08** | **1.37** | **-0.74^a^** | **1.29** | **0.008** | **0.04** |
| lh_precuneus_vol | -0.26 | 1.13 | 0.06 | 1.12 | -0.67 | 1.60 | 0.50 | 2.10 | 0.45 | 0.70 | -0.42 | 1.38 | -0.33 | 1.46 | -0.29 | 1.13 | 0.38 | 1.50 | 0.32 | 0.50 |
| lh_rostralanteriorcingulate_vol | -0.16 | 1.10 | -0.31 | 1.12 | -0.79 | 0.87 | 0.97 | 2.06 | 0.06 | 0.52 | -0.56 | 1.04 | -0.41 | 0.92 | 0.09 | 1.53 | -0.23 | 1.25 | 0.54 | 0.68 |
| lh_rostralmiddlefrontal_vol | -0.43 | 0.92 | -0.39 | 0.96 | -0.81 | 1.11 | -0.06 | 1.64 | 0.56 | 0.79 | -0.54 | 1.03 | -0.60 | 0.73 | -0.08 | 1.21 | -0.79 | 1.07 | 0.35 | 0.50 |
| lh_superiorfrontal_vol | -0.08 | 0.76 | -0.05 | 0.59 | -0.02 | 1.16 | -0.41 | 0.65 | 0.68 | 0.81 | 0.17 | 0.91 | -0.23 | 0.63 | 0.13 | 0.76 | -0.49 | 0.78 | 0.03 | 0.10 |
| lh_superiorparietal_vol | -0.30 | 1.03 | 0.27 | 0.92 | -0.27 | 0.97 | 0.24 | 1.70 | 0.23 | 0.65 | -0.35 | 1.02 | -0.10 | 1.41 | -0.11 | 0.90 | 0.41 | 0.82 | 0.15 | 0.37 |
| lh_superiortemporal_vol | -0.17 | 0.50 | -0.38 | 0.87 | -0.80 | 1.49 | -1.07 | 1.11 | 0.06 | 0.52 | **-0.09** | **0.92** | **-0.98** | **1.36** | **-0.16^d^** | **0.61** | **-0.79^c^** | **0.86** | **0.007** | **0.04** |
| lh_supramarginal_vol | 0.01 | 1.29 | -0.12 | 0.86 | 0.51 | 1.22 | -0.40 | 2.19 | 0.35 | 0.70 | 0.36 | 1.22 | 0.48 | 0.94 | -0.15 | 1.16 | -0.47 | 1.51 | 0.21 | 0.43 |
| lh_temporalpole_vol | 0.09 | 1.14 | -0.23 | 1.35 | -0.10 | 0.78 | -0.43 | 0.74 | 0.42 | 0.70 | -0.39 | 0.97 | -0.02 | 0.99 | -0.07 | 1.16 | 0.06 | 1.34 | 0.69 | 0.81 |
| lh_transversetemporal_vol | -0.25 | 0.64 | -0.39 | 0.98 | -0.44 | 0.93 | -0.23 | 0.62 | 0.67 | 0.81 | -0.09 | 0.81 | -0.36 | 0.79 | -0.42 | 0.90 | -0.50 | 0.79 | 0.53 | 0.68 |
| rh_bankssts_vol | -0.27 | 1.12 | -0.21 | 1.10 | -0.65 | 1.15 | -0.42 | 1.55 | 0.77 | 0.84 | -0.53 | 1.05 | -0.99 | 1.05 | -0.13 | 1.10 | 0.21 | 1.15 | 0.02 | 0.07 |
| rh_caudalanteriorcingulate_vol | 0.01 | 0.91 | 0.03 | 0.98 | 0.20 | 0.79 | -0.11 | 1.30 | 0.94 | 0.94 | 0.31 | 0.89 | 0.22 | 1.01 | -0.03 | 0.99 | -0.29 | 0.76 | 0.09 | 0.18 |
| rh_caudalmiddlefrontal_vol | 0.30 | 1.11 | 0.02 | 1.24 | 0.55 | 1.14 | -0.50 | 1.08 | 0.28 | 0.65 | 0.43 | 0.86 | 0.15 | 1.49 | 0.40 | 1.13 | -0.29 | 1.12 | 0.28 | 0.38 |
| rh_cuneus_vol | 0.01 | 0.62 | -0.004 | 0.71 | 0.11 | 0.93 | -0.21 | 0.83 | 0.78 | 0.84 | 0.28 | 0.60 | -0.01 | 0.73 | 0.03 | 0.79 | -0.29 | 0.76 | 0.11 | 0.20 |
| rh_entorhinal_vol | -0.21 | 0.89 | -0.80 | 1.14 | -0.53 | 1.01 | -0.23 | 0.74 | 0.34 | 0.65 | -0.53 | 0.90 | -0.87 | 1.17 | -0.27 | 0.88 | -0.36 | 1.11 | 0.33 | 0.43 |
| rh_frontalpole_vol | 0.16 | 1.33 | -0.20 | 1.18 | 0.36 | 0.95 | -0.32 | 1.38 | 0.35 | 0.65 | 0.04 | 1.32 | -0.26 | 1.05 | 0.19 | 1.33 | 0.17 | 1.08 | 0.82 | 0.87 |
| rh_fusiform_vol | 0.16 | 1.59 | 0.50 | 1.08 | 0.09 | 1.34 | -0.33 | 1.34 | 0.31 | 0.65 | **0.36** | **1.19** | **1.07^d^** | **1.41** | **-0.02** | **1.27** | **-0.50^b^** | **1.11** | **0.01** | **0.04** |
| rh_inferiorparietal_vol | -0.24 | 0.80 | -0.07 | 0.59 | -0.18 | 1.01 | -0.65 | 0.55 | 0.19 | 0.65 | 0.09 | 0.58 | -0.13 | 0.63 | -0.24 | 0.79 | -0.55 | 0.97 | 0.11 | 0.20 |
| rh_inferiortemporal_vol | -0.49 | 1.15 | -0.85 | 1.39 | -0.64 | 1.29 | -0.42 | 0.91 | 0.57 | 0.84 | -0.82 | 1.09 | -1.25 | 1.48 | -0.49 | 1.05 | -0.05 | 1.14 | 0.06 | 0.14 |
| rh_insula_vol | -0.18 | 0.60 | -0.06 | 0.65 | -0.23 | 0.79 | -0.74 | 0.83 | 0.24 | 0.65 | 0.13 | 0.57 | -0.22 | 0.77 | -0.34 | 0.73 | -0.36 | 0.65 | 0.08 | 0.17 |
| rh_isthmuscingulate_vol | -0.09 | 1.04 | -0.31 | 0.98 | -0.06 | 1.28 | 0.54 | 1.08 | 0.32 | 0.65 | -0.27 | 1.06 | -0.16 | 1.07 | 0.03 | 1.25 | -0.03 | 0.97 | 0.96 | 0.96 |
| rh_lateraloccipital_vol | 0.04 | 0.73 | -0.01 | 0.85 | 0 | 1.02 | -0.46 | 1.03 | 0.64 | 0.84 | 0.17 | 0.88 | -0.17 | 0.79 | 0.31 | 0.78 | -0.53 | 0.83 | 0.02 | 0.07 |
| rh_lateralorbitofrontal_vol | 0.20 | 1.13 | 0.56 | 1.21 | 0.47 | 1.36 | -0.87 | 1.27 | 0.09 | 0.65 | 0.17 | 1.19 | 0.51 | 1.52 | 0.41 | 1.24 | 0.05 | 1.16 | 0.50 | 0.63 |
| rh_lingual_vol | -0.07 | 0.73 | -0.05 | 1.04 | 0.19 | 1.01 | -0.25 | 0.70 | 0.78 | 0.84 | 0.02 | 0.72 | -0.07 | 1.04 | 0.18 | 0.99 | -0.25 | 0.85 | 0.56 | 0.65 |
| rh_medialorbitofrontal_vol | -0.08 | 0.93 | -0.35 | 1.04 | -0.06 | 1.09 | 0.38 | 0.93 | 0.42 | 0.74 | 0.11 | 1.04 | -0.33 | 0.90 | -0.25 | 1.18 | -0.02 | 0.82 | 0.58 | 0.65 |
| rh_middletemporal_vol | -0.26 | 0.68 | -0.51 | 0.76 | -0.58 | 0.99 | -0.68 | 0.81 | 0.65 | 0.84 | -0.34 | 0.74 | -0.81 | 0.85 | -0.11 | 0.67 | -0.69 | 0.80 | 0.04 | 0.09 |
| rh_paracentral_vol | -0.04 | 0.89 | 0.11 | 0.86 | 0.46 | 0.83 | -0.37 | 0.85 | 0.09 | 0.65 | 0.45 | 0.60 | 0.001 | 0.81 | -0.05 | 0.72 | 0.02 | 1.27 | 0.04 | 0.09 |
| rh_parahippocampal_vol | -0.06 | 1.13 | -0.30 | 1.21 | -0.38 | 1.03 | 0.36 | 1.01 | 0.29 | 0.65 | -0.18 | 1.11 | -0.71 | 1.11 | 0.11 | 1.17 | -0.05 | 0.99 | 0.16 | 0.25 |
| rh_parsopercularis_vol | 0.11 | 1.11 | 0.02 | 0.59 | -0.14 | 0.88 | -0.49 | 0.64 | 0.28 | 0.65 | **0.26^d^** | **0.58** | **-0.04** | **0.66** | **0.15** | **1.10** | **-0.59^a^** | **0.75** | **0.002** | **0.02** |
| rh_parsorbitalis_vol | -0.07 | 0.86 | 0.17 | 0.93 | 0.28 | 1.20 | -0.01 | 1.07 | 0.79 | 0.84 | 0.06 | 1.15 | 0.21 | 1.06 | -0.03 | 0.93 | 0.19 | 0.81 | 0.68 | 0.75 |
| rh_parstriangularis_vol | -0.01 | 0.88 | -0.25 | 0.85 | -0.30 | 0.97 | -0.47 | 0.47 | 0.56 | 0.84 | 0.12 | 0.64 | -0.37 | 0.94 | -0.01 | 0.81 | -0.62 | 0.91 | 0.01 | 0.07 |
| rh_pericalcarine_vol | 0.04 | 0.83 | -0.21 | 1.22 | -0.17 | 1.00 | -0.58 | 1.07 | 0.59 | 0.84 | 0.31 | 0.66 | -0.35 | 0.89 | -0.09 | 1.35 | -0.55 | 0.87 | 0.02 | 0.07 |
| rh_postcentral_vol | -0.16 | 0.83 | 0.02 | 0.64 | 0.32 | 0.85 | -0.30 | 0.61 | 0.046 | 0.65 | 0.42 | 0.75 | 0.03 | 0.46 | -0.09 | 0.72 | -0.35 | 0.93 | 0.03 | 0.09 |
| rh_posteriorcingulate_vol | -0.13 | 1.19 | -0.11 | 1.13 | -0.33 | 1.67 | 0.60 | 1.69 | 0.64 | 0.84 | -0.21 | 1.41 | -0.32 | 1.29 | -0.27 | 1.44 | 0.44 | 1.13 | 0.20 | 0.28 |
| rh_precentral_vol | 0.04 | 1.95 | 0.45 | 1.56 | 0.49 | 1.69 | -0.44 | 0.87 | 0.13 | 0.65 | 0.49 | 0.88 | 0.36 | 1.90 | -0.17 | 1.25 | 0.39 | 2.48 | 0.17 | 0.25 |
| rh_precuneus_vol | -0.52 | 1.56 | -0.09 | 1.06 | -0.43 | 1.24 | -0.04 | 1.19 | 0.78 | 0.84 | -0.14 | 1.20 | -0.46 | 1.13 | -0.39 | 1.69 | -0.24 | 0.99 | 0.87 | 0.90 |
| rh_rostralanteriorcingulate_vol | -0.20 | 0.96 | -0.28 | 0.89 | -0.37 | 0.95 | 0.001 | 1.49 | 0.89 | 0.92 | -0.41 | 1.11 | -0.59 | 0.94 | 0.13 | 0.84 | -0.22 | 0.91 | 0.16 | 0.25 |
| rh_rostralmiddlefrontal_vol | 0.24 | 1.16 | -0.16 | 1.24 | -0.37 | 1.38 | -0.77 | 1.13 | 0.28 | 0.65 | **0.34^d^** | **0.56** | **-0.46** | **1.37** | **0.26** | **1.28** | **-0.82^a^** | **1.34** | **0.001** | **0.01** |
| rh_superiorfrontal_vol | -0.04 | 1.38 | -0.08 | 0.69 | 0.07 | 1.86 | -0.59 | 1.16 | 0.21 | 0.65 | **0.55^b,d^** | **0.67** | **-0.20^a^** | **0.75** | **0.26^d^** | **1.03** | **-1.07^a,c^** | **1.92** | **0.0001** | **0.005** |
| rh_superiorparietal_vol | -0.56 | 1.10 | 0.18 | 0.74 | 0.01 | 1.22 | 0.20 | 1.18 | 0.05 | 0.65 | -0.12 | 0.93 | -0.14 | 0.99 | -0.33 | 1.27 | 0.26 | 0.97 | 0.53 | 0.64 |
| rh_superiortemporal_vol | -0.10 | 0.50 | -0.16 | 0.58 | -0.09 | 1.10 | -0.53 | 0.56 | 0.43 | 0.74 | 0.10 | 0.80 | -0.42 | 0.58 | 0.02 | 0.73 | -0.43 | 0.59 | 0.04 | 0.09 |
| rh_supramarginal_vol | 0.02 | 0.97 | 0.12 | 0.98 | 0.75 | 1.87 | -0.93 | 1.65 | 0.12 | 0.65 | 0.45 | 0.75 | 0.26 | 1.23 | -0.06 | 1.07 | -0.03 | 2.14 | 0.12 | 0.21 |
| rh_temporalpole_vol | 0.06 | 1.31 | -0.68 | 1.46 | 0.06 | 0.86 | 0.22 | 1.78 | 0.17 | 0.65 | -0.65 | 0.85 | -0.44 | 1.78 | -0.005 | 1.50 | 0.39 | 0.83 | 0.02 | 0.07 |
| rh_transversetemporal_vol | -0.07 | 0.59 | 0.10 | 1.04 | 0.30 | 1.16 | -0.08 | 0.41 | 0.77 | 0.84 | **0.67^c,d^** | **1.01** | **0.12** | **0.68** | **-0.18^a^** | **0.86** | **-0.31^a^** | **0.72** | **0.004** | **0.03** |

**eTable 4.** Number of available observations for clinical assessments and cognitive composite scores over the follow-up period.

Abbreviations: FU, follow-up (in years); Alzheimer’s disease spectrum, ADS; Hippocampal-sparing, HpSp; Limbic-predominant, LP; MMSE, mini-mental-state examination; CDR, clinical dementia ratio; MEM, memory; EF, executive function; LAN, language; VIS, visuospatial functioning

|  |  | Tau clusters | | | | Atrophy clusters | | | | ADS (n=166) | Healthy conrols (n=154) |
| --- | --- | --- | --- | --- | --- | --- | --- | --- | --- | --- | --- |
|  |  | Posterior (N=57) | Limbic (N=53) | MTL-sparing (N=36) | Left temporal (N=20) | HpSp (N=32) | Diffuse (N=58) | Minimal atrophy (N=41) | LP (N=35) |  |  |
| BL | MMSE | 56 | 52 | 35 | 20 | 32 | 57 | 40 | 34 | 163 | 146 |
|  | CDR-SoB | 56 | 52 | 35 | 20 | 32 | 57 | 40 | 34 | 163 | 147 |
|  | MEM | 57 | 52 | 35 | 20 | 32 | 57 | 41 | 34 | 164 | 152 |
|  | EF | 57 | 52 | 35 | 20 | 32 | 57 | 41 | 34 | 164 | 152 |
|  | LAN | 57 | 52 | 35 | 20 | 32 | 57 | 41 | 34 | 164 | 152 |
|  | VIS | 57 | 52 | 35 | 20 | 32 | 57 | 41 | 34 | 164 | 152 |
| FU-1 | MMSE | 39 | 30 | 26 | 18 | 26 | 39 | 26 | 22 | 113 | 36 |
|  | CDR-SoB | 43 | 37 | 26 | 18 | 27 | 41 | 32 | 24 | 124 | 36 |
|  | MEM | 43 | 36 | 27 | 18 | 28 | 41 | 31 | 24 | 124 | 39 |
|  | EF | 43 | 36 | 27 | 18 | 28 | 41 | 31 | 24 | 124 | 39 |
|  | LAN | 43 | 36 | 27 | 18 | 28 | 41 | 31 | 24 | 124 | 39 |
|  | VIS | 43 | 36 | 27 | 18 | 28 | 41 | 31 | 24 | 124 | 39 |
| FU-2 | MMSE | 28 | 25 | 17 | 9 | 15 | 24 | 24 | 16 | 79 | 92 |
|  | CDR-SoB | 34 | 27 | 17 | 11 | 15 | 29 | 25 | 20 | 89 | 103 |
|  | MEM | 27 | 27 | 20 | 9 | 17 | 24 | 25 | 17 | 83 | 98 |
|  | EF | 27 | 27 | 20 | 9 | 17 | 24 | 25 | 17 | 83 | 98 |
|  | LAN | 27 | 27 | 20 | 9 | 17 | 24 | 25 | 17 | 83 | 98 |
|  | VIS | 27 | 27 | 20 | 9 | 17 | 24 | 25 | 17 | 83 | 98 |
| FU-3 | MMSE | 22 | 18 | 16 | 4 | 12 | 11 | 20 | 17 | 60 | 19 |
|  | CDR-SoB | 24 | 24 | 18 | 4 | 14 | 15 | 23 | 18 | 70 | 26 |
|  | MEM | 22 | 17 | 14 | 5 | 11 | 11 | 21 | 15 | 58 | 19 |
|  | EF | 22 | 17 | 14 | 5 | 11 | 11 | 21 | 15 | 58 | 19 |
|  | LAN | 22 | 17 | 14 | 5 | 11 | 11 | 21 | 15 | 58 | 19 |
|  | VIS | 22 | 17 | 14 | 5 | 11 | 11 | 21 | 15 | 58 | 19 |
| FU-4 | MMSE | 13 | 10 | 6 | 3 | 8 | 4 | 13 | 7 | 32 | 69 |
|  | CDR-SoB | 13 | 11 | 6 | 3 | 8 | 6 | 11 | 8 | 33 | 69 |
|  | MEM | 9 | 9 | 7 | 2 | 6 | 4 | 9 | 8 | 27 | 67 |
|  | EF | 9 | 9 | 7 | 2 | 6 | 4 | 9 | 8 | 27 | 67 |
|  | LAN | 9 | 9 | 7 | 2 | 6 | 4 | 9 | 8 | 27 | 67 |
|  | VIS | 9 | 9 | 7 | 2 | 6 | 4 | 9 | 8 | 27 | 67 |
| FU-5 | MMSE | 4 | 4 | 2 | 1 | 3 | 3 | 2 | 3 | 11 | 16 |
|  | CDR-SoB | 6 | 5 | 2 | 1 | 3 | 4 | 3 | 4 | 14 | 20 |
|  | MEM | 2 | 4 | 2 | 1 | 4 | 1 | 2 | 2 | 9 | 11 |
|  | EF | 2 | 4 | 2 | 1 | 4 | 1 | 2 | 2 | 9 | 11 |
|  | LAN | 2 | 4 | 2 | 1 | 4 | 1 | 2 | 2 | 9 | 11 |
|  | VIS | 2 | 4 | 2 | 1 | 4 | 1 | 2 | 2 | 9 | 11 |
